# Soil‐Borne Pathogens Reflect Agricultural Land‐Use Legacies

**DOI:** 10.1111/ele.70332

**Published:** 2026-02-11

**Authors:** Tord Ranheim Sveen, Ida Junker Madsen, Eva Gustavsson, Sara Cousins, Franz Buegger, Karin Pritsch, Laura Riggi, Janne Bengtsson, Maria Viketoft, Mo Bahram

**Affiliations:** ^1^ Department of Ecology Swedish University of Agricultural Sciences Uppsala Sweden; ^2^ Department of Agroecology Aarhus University Slagelse Denmark; ^3^ Department of Conservation University of Gothenburg Mariestad Sweden; ^4^ Department of Physical Geography Stockholm University Stockholm Sweden; ^5^ Research Unit Environmental Simulation, Helmholtz Zentrum München, German Research Center for Environmental Health Neuherberg Germany; ^6^ Environmental Research, Wageningen University and Research Wageningen the Netherlands; ^7^ Institute of Ecology and Earth Sciences, University of Tartu Tartu Estonia

**Keywords:** land‐use change, legacy decay, legacy effects, pathogens, soil microbes

## Abstract

Historical land‐use changes shape present‐day biodiversity through legacy effects, but the duration and mechanisms of these legacies are poorly understood. We used historical land‐use maps in two Swedish landscapes across three centuries to examine the persistent influence of historical land use on plant and soil microbial communities. Overall, bacteria showed stronger legacy effects than fungi, but effects varied across functional groups of plant‐associated and free‐living taxa. However, soil‐borne plant pathogenic fungi showed a persisting influence of arable land use which gradually disappeared after ~150 years, suggesting that land‐use legacies decay over time. This dilution could relate to changing plant communities but also to changes in microbial associations, as suggested by species co‐occurrence patterns over time. Our findings provide novel and crucial information on the duration of land‐use legacies and single out soil‐borne plant pathogens as key indicator groups of historical land use in present‐day ecosystems.

## Graphical abstract

Legacy effects profoundly shape the distribution and diversity of terrestrial communities, but are difficult to grasp over longer timescales. Here, we use historical land‐use maps to study lingering legacies of historical land use on present‐day microbial communities. Our findings show that soil‐borne fungal pathogens are key groups to follow as indicators of historical arable land use.

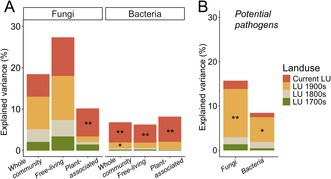

## 1 Introduction

Landscapes and ecosystems are embedded in historical contingencies that continue to influence current biodiversity patterns and processes (Ingold 1993) but understanding the causes, strength and duration of these historical legacies remains a key challenge in ecology (Foster et al. 2003). Broadly defined as the persisting influence attributed to factors after their direct causal interactions have ceased (Wurst and Ohgushi 2015), legacy effects can extend over centuries or even millennia (Dupouey et al. 2002) and be attributed to a number of mechanisms, from environmental effects to alterations of resources, habitats and species interactions (Perring et al. 2016; Vass and Langenheder 2017).

Persisting effects of historical land use on present‐day communities is arguably the most well‐studied area of legacy effects. Historical land use has been found to predict the current diversity of both grassland plants and soil microbes better than present‐day land use (Auffret et al. 2018; Gustavsson et al. 2007; Jangid et al. 2011; Midolo et al. 2025), usually attributed to irreversible modifications of soil abiotic properties like pH, carbon and nitrogen (Dupouey et al. 2002; Saatkamp et al. 2021), but also to landscape‐level land use in the past (Lindborg and Eriksson 2004). These land‐use legacies can be of concrete importance for ecosystem functions and functioning (Bürgi et al. 2017), but also for conservation. For instance, studies involving historical land‐use maps moreover show that plant specialists associated with specific grassland management practices persist in habitats outside their preferred niche range decades or even centuries after their preferred habitat changed (Auffret et al. 2018; Gustavsson et al. 2007). Deeper knowledge about how long these ‘ghost populations’ remain, and by which mechanisms they persist, is of high value to mitigate their possible extinction (Tilman et al. 1994). Increasing interest in incorporating soil legacies to aid both plant restoration and production (Albertson et al. 2024; Bakker et al. 2018; Brinkman et al. 2017; Weidlich et al. 2021) is nevertheless restricted by the lack of adequate source material to infer historical land use (Bürgi et al. 2017). The temporal extension and functional differentiations of land‐use legacies within soil microbial communities is also largely unknown, despite their intrinsic links to plant communities aboveground.

Given the key role of soil organisms in nutrient cycling and overall ecosystem functioning, understanding their response to land‐use changes is critically important. For example, Tsiafouli et al. (2015) found large negative effects of agricultural management intensity on the richness of several soil animal groups, usually affecting higher trophic levels more. Arable farming and land‐use intensification may be negative for fungi (Liu et al. 2023; Turley et al. 2020; Vahter et al. 2022), but favour bacteria (e.g., Labouyrie et al. 2023) as these generally respond faster and stronger to land‐use changes (Cline and Zak 2015; Sveen et al. 2024). Yet, despite evidence that land‐use legacies may be predominantly encoded in the relative balance between beneficial and pathogenic taxa rather than the whole microbial community (Bennett and Klironomos 2019), studies examining functionally differentiated responses between, for example, free‐living and plant‐associated microbial groups are missing. Plant pathogens (hereafter pathogens) could be an especially important indicator of historical land use since these typically accumulate during management intensification (e.g., Le Provost et al. 2020) but we have no corresponding knowledge about their subsequent disappearance after extensification or land‐use change.

Here, we used historical land‐use maps from two Swedish landscapes where land use and land‐use changes have been documented for more than 200 years, back to the 18th century (Cousins 2001; Gustavsson et al. 2007), to examine the persistence of land‐use legacy effects on present‐day soil microbial communities and assess the pathways that may cause their eventual disappearance. The specific land use changes examined were traditional arable to grasslands from the 1800s and 1900s, and grasslands to forests from the 1900s onwards. Past arable management was low‐intensive crop rotations, sometimes including fallows, and grasslands were mainly managed by grazing with low or no fertilisation, that is, traditional management. We hypothesized that land‐use legacies (i) are stronger (i.e., more persistent) on the community structure of bacteria than fungi, due to their closer and more immediate interactions with soil abiotic properties affected by land‐use changes; (ii) are stronger on plant‐associated taxa than free‐living; with tangible legacies of arable land use on soil‐borne pathogens; and (iii) dilute and gradually diminish over time for all microbial groups.

## 2 Methods

### 2.1 Site Description and Soil Sampling

**FIGURE 1:**
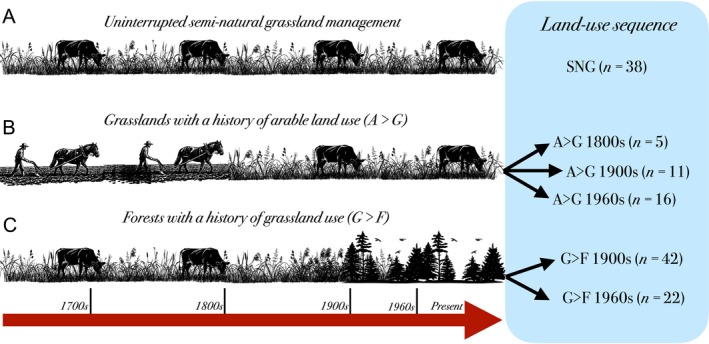
Historical land use and land‐use sequences in the study regions. The two study regions examined are characterised by three main types of land‐use and land‐use change. Sites characterised by continuous semi‐natural grassland management (A), which formed the historical backbone of central‐Swedish agriculture up until the mid‐20th century but which has subsequently declined drastically; sites historically managed as arable fields (B) but which have been converted to grasslands (Arable‐to‐Grassland; A>G) at some point in history; and sites historically managed as grasslands (C) but which have been abandoned and afforested during the 20th century (Grassland‐to‐Forest; G>F). The sites in (B, C) were further classified into five different land‐use sequences depending on their respective time of land‐use conversion, whereas the sequence comprising sites with uninterrupted semi‐natural grassland management (SNG) was used as a baseline to examine legacy effects.

We sampled 134 sites from two regions in southern Sweden (Nynäs, Källtorp) characterised by long histories of livestock grazing and haymaking in addition to arable farming (Cousins et al. 2002). Full details on climate and geography for each respective region can be found in Table S1 and Supporting Information, including a brief overview of the kind of agriculture and management practices conducted historically. All sites have been previously classified according to historical and present‐day land use at five differing time points based on cadastral maps and aerial photographs: 1700s, 1800s, 1900s, 1960s and current landuse (Cousins 2001; Gustavsson et al. 2007). Next, we constructed land‐use sequences (LUS) based on the time of conversion between differing land uses over the time period examined, that is, 1700s to the present day, following the method established in Gustavsson et al. (2007). Briefly, there were three dominating land uses and land‐use changes in the study regions: sites characterised by uninterrupted semi‐natural grassland management (SNG; Figure 1A); sites with a history of arable management but which have been subsequently converted to grasslands at different time points (A>G; Figure 1B); and sites with a history of grassland management which have been abandoned and afforested (grassland‐to‐forest, G>F; Figure 1C). This resulted in a total of six different land‐use sequences based on the type and time of land‐use change (Table S2). Legacy effects were evaluated by comparing sites with contrasting histories (i.e., sites with differing LUS) under the explicit assumption that consistent differences between sites of similar present‐day but different historical land use is due to legacies stemming from the latter (Bürgi et al. 2017). In other words, we assume that the duration of legacy effects can be discerned from the time since land‐use change when sites share current land use. Crucially, as sites with arable land use were converted to grasslands characterised by SNG management at differing time points, we used uninterrupted SNGs (Figure 1A) as the baseline for inferring legacy signals. While we acknowledge that this approach draws heavily on space‐for‐time substitutions, where confounding site‐level differences cannot be corrected for in any meaningful way (Johnson and Miyanishi 2008), time‐series tracking community development over centurial timescales are virtually inexistent and thus leave few practical alternatives.

The sites were sampled during the period July–September 2021, using a soil corer (diameter = 3 cm, depth = 10 cm) and by pooling multiple subsamples into a composite sample representative of the site. Further details on the soil sampling and sample processing prior to molecular handling and physico‐chemical characterisation can be found in the Supporting Information.

### 2.2 Plant Survey

Contemporary land‐cover maps were used as a baseline for field investigations, in which plant species richness and cover (%) of all vascular plants and ferns down to species level was investigated in 5 × 5 m plots at all sites. These surveys were conducted in 2015 (Nynäs) and 2021 (Källstorp). Previous studies have shown that plot‐level species richness adequately captures total species richness at spatial scales up to 1–5 ha (Lindborg and Eriksson 2004; Öster et al. 2007), with little temporal variation over time when management is kept constant. Plants were additionally classified into the following functional type categories: Forbs, graminoids, ferns and woody plants.

### 2.3 Molecular Analyses & Bioinformatics

DNA was extracted from ~200 mg of frozen soil and root samples using the PowerMax Soil DNA Isolation Mini kit (Qiagen GmbH, Hilden, Germany) following the manufacturer's instructions. All amplicons were sequenced on Illumina MiSeq 2500 platforms. We used the LotuS2 version 2.22 (Özkurt et al. 2022) pipeline to quality‐filter, demultiplex and process the filtered reads into operational taxonomic units (OTUs) for fungi, and amplicon sequence variants (ASV) for bacteria. For full details on the wet lab and bioinformatic processing used for metabarcoding, we refer the reader to the Supporting Information (SM). This yielded an average of 50,492 (sd = 19,244) bacterial and 15,686 (sd = 8873) fungal sequences per sample. Rarefaction curves showed adequate sequencing depth for most samples (Figure S1) except for one fungal sample which was discarded due to insufficient sequencing depth (< 3000 reads). The remaining communities (*n* = 133, sequencing depth = 3680 for fungi, *n* = 134, sequencing depth = 13,376 for bacteria) were rarefied to minimum sequencing depth prior to alpha and beta diversity analyses. To ensure that our findings were not affected by sequencing depth, we additionally ran all analyses on raw (i.e., unrarefied) matrices in parallel. As our findings were consistent regardless of standardisation (Figures S5 and S6), we present only the results from the rarefied matrices. The raw sequences have been deposited at NCBI under accession PRJNA1238174.

### 2.4 Categorization of Microbial Groups

We annotated microbial taxa into categories of free‐living, plant‐associated and plant‐pathogenic based on public databases. Plant‐associated fungi were designated based on the *FungalTraits* database (ver. 1.2 (Põlme et al. 2020)) and included taxa assigned as mycorrhizal fungi (arbuscular‐ and ectomycorrhizal fungi) and plant pathogens. This yielded a total of 697 plant‐associated fungal OTUs, representing 16.3% of all fungal OTUs across 187 genera, out of which 254 OTUs across 124 genera were classified as plant pathogens.

For bacteria, we used the recently assembled database of plant‐associated bacteria published in Li et al. (2023). All ASVs annotated as either ‘plant‐growth promoting’ or ‘phytopathogenic’ were considered plant‐associated. These comprised 1467 taxa (8.3% of all taxa) across 150 genera. As many plant‐associated bacteria exhibit opposite pathogenic and growth‐promoting capacities at strain level (Santoyo et al. 2012), which are not captured through amplicon sequencing, we assigned the status of ‘potential pathogen’ to bacterial taxa while noting that these may in fact have the opposite growth‐promoting effects. Potential bacterial pathogens comprised 465 taxa across 29 genera.

### 2.5 Statistical Analyses

#### 2.5.1 Legacy Effects of Historical Land Use on Microbial Diversity

We used a two‐step approach to assess the influence of historical land use on present‐day diversity of microbial communities. Firstly, richness was regressed as a response variable against factorial variables describing each site's land‐use history (e.g., Richness~LU 1700 + LU 1800 + LU 1900 + LU current) using generalised linear models (GLM). The significance of each factor was then evaluated using omnibus ANOVA Type II tests, and the explained variance of each factor was partitioned using the *glmm.hp* package (Lai et al. 2022). GLMs were also used to discriminate legacy effects between differing land‐use sequences. In line with previous studies on plant populations in the study regions (Gustavsson et al. 2007), we opted for using sites with a history of uninterrupted semi‐natural grassland management (i.e., SNG) as the baseline for comparisons. Briefly, richness was regressed against each land‐use sequence (e.g., Richness ~ LUS), with SNG sites as reference levels and an interaction term with region to see if responses varied with geographic region. All statistical analyses were conducted using *R* (ver. 4.4.3) (R Core Team 2022). Tests involving multiple comparisons were adjusted using Benjamin–Hochberg's corrected *p*‐values.

#### 2.5.2 Compositional Changes, Indicator Taxa and Links Between Plant–Soil Communities

We used permutational multivariate analyses (*adonis2* from the *vegan* package (Oksanen et al. 2022)) to test for differences in community composition based on Bray–Curtis and Jaccard distances for microbial and plant communities, respectively. We also identified indicator taxa of plants and pathogenic microbes specific to the differing LUS using the *multipatt* function in the *indicspecies* package (Cáceres and Legendre 2009). Tests for differences in the relative abundances of microbial indicator genera between LUS were done using the ANCOM‐BC method (Lin and Peddada 2024). Spearman correlations were used to assess links between plant and microbial community richness and composition.

#### 2.5.3 Examination of Changes in Microbial Communities With Co‐Occurrence Analysis

We constructed microbial co‐occurrence networks for each land‐use sequence based on pairwise correlations between OTUs to infer the potential for interactions between co‐occurring taxa using the *igraph* package (Csárdi et al. 2025). More details of the network construction are found in the Supporting Information.

## 3 Results

### 3.1 Plant and Soil Legacies

We found overall few differences in soil properties between the differing land‐use sequences (LUS), except an increasing C:N ratio in abandoned and afforested grasslands (Table S4). Notably, sites with a history of arable land use did not differ from the baseline of semi‐natural grassland sites in any of the soil properties examined (Kruskal–Wallis, *p* > 0.05 for all tests).

**FIGURE 2:**
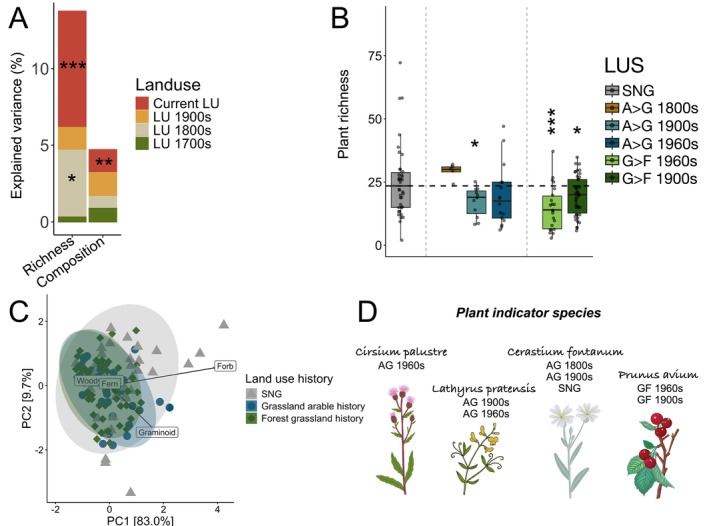
Plant community diversity and composition as affected by historical land‐use changes. (A) The proportion (Explained variance, *R* ^*2*^) of present‐day plant richness and community composition attributed to current and historical land use (LU). (B) Present‐day plant richness across sites classified into six differing land‐use sequences (LUS) based on their respective land‐use history and time since land‐use change (Table S2). (C) PCoA of plant community composition across the differing LUS. (D) A select few indicator plant species associated with differing LUS. AG denotes land‐use change from arable to grassland, GF denotes land‐use change from grassland to forest. Asterisks (*) denote significant influence of land‐use factor to present‐day biodiversity based on GLMs with the following significance levels: *p < 0.05, **p < 0.01, ***p < 0.001. In panel B, semi‐natural grasslands (SNG) are set as the baseline for comparisons, with the dashed horizontal line indicating the median plant richness of SNG sites. Detailed test results for GLMs, Permanovas and indicator species analyses can be found in Tables S5–S7.

Present‐day plant richness was related to current and historical (1800s) land use, whereas community composition was significantly related only to current land use (Figure 2A). Plant richness was moreover lower in sites with a history of arable land use in the 1900s than in semi‐natural grasslands (GLM, *t* = −2.29, *p* = 0.024). Grassland abandonment and afforestation also led to decreased plant richness compared to the SNG baseline (Figure 2A; Table S5). Plant community composition differed between current and abandoned grasslands (Permanova, *F* = 2.08, *p* = 0.02; Table S6), underpinned by a shift from forbs to graminoid species (Figure 2C). Indicator species analyses singled out a total of nine plant species as specific to grassland sites with an arable history (Table S7). In particular, *Trifolium pratense* and *Juncus effusus* were designated indicators of all land‐use sequences comprising arable land use, whereas *Prunus avium* was conversely singled out as an indicator of afforested grasslands (Table S7; Figure 2D). No indicator plant was exclusively associated with uninterrupted semi‐natural grassland management.

### 3.2 Bacterial and Fungal Legacy Effects Vary Depending on Community and Functional Group

**FIGURE 3:**
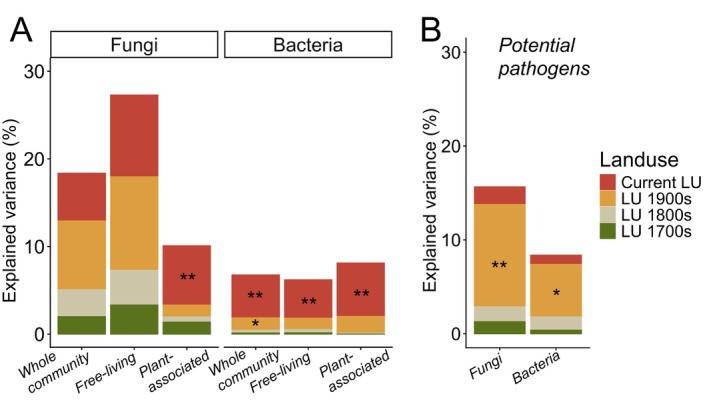
Contribution of historical and current land use to present‐day microbial diversity. (A) The proportion (Explained variance, *R* ^2^) of present‐day microbial diversity attributed to current and historical land use (LU) across fungal and bacterial communities and their fractions of free‐living and plant‐associated taxa. In panel (B) corresponding effects on beneficial and pathogenic taxa are shown. Asterisks (*) denote significant influence of land‐use factor to present‐day biodiversity based on GLMs with the following significance levels: *p < 0.05, **p < 0.01, ***p < 0.001. Full test details are found in Table S8.

Land‐use legacies continued to shape the diversity and composition of present‐day bacterial but not fungal communities. Specifically, both present‐day diversity and community composition of whole bacterial communities were significantly related to both historical (1900s) and current land use (Figures 2A and S3A; Tables S8 and S9), and this was true also for the composition of free‐living and plant‐associated taxa (Figure S3A). By contrast, fungal groups were either associated only to current land use (richness of plant‐associated fungi, composition of all groups), or not associated to any land use at all (Figures 3A and S3A). Yet, the strongest influence of historical land use was found at the level of pathogenic fungi and potentially pathogenic bacteria. Here, historical land use exerted a strong influence especially on fungal pathogens, and the richness of both bacterial and fungal pathogens was related exclusively to historical rather than current land use (Figure 2B; Table S8). Historical influence moreover stretched back to the 1800s for fungal pathogen composition (Figure S3B).

### 3.3 Compositional Analyses Reveal Genera‐Specific Pathogen Legacies

**FIGURE 4:**
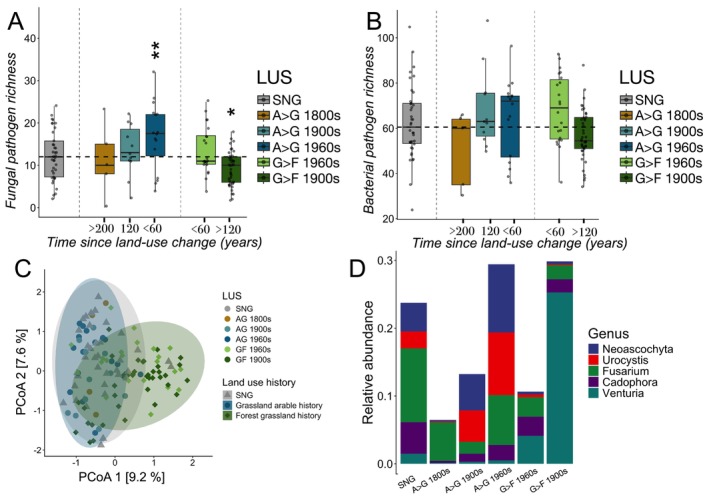
Legacies of arable land use in the present‐day richness and composition of soil‐borne pathogens. Present‐day richness of fungal (A) and bacterial (B) soil‐borne pathogens based on their respective land‐use history and time since land‐use change (Table S2). Asterisks (*) denote significant influence of land‐use factor to present‐day biodiversity based on GLMs with the following significance levels: *p < 0.05, **p < 0.01, ***p < 0.001. The dashed horizontal line indicates median pathogen richness of the baseline SNG sites. (C) PCoA of fungal pathogen community composition across the differing land‐use sequences. (D) Relative abundances of five key fungal pathogenic genera of differing land‐use histories. *Neoascochyta* and *Urocystis* are indicator genera for present‐day grasslands with a history of arable land use. *Fusarium* is indicator genus of present‐day grasslands, independent of arable land use history. *Venturia* indicates forests with a grassland history. Detailed test results for GLMs, Permanovas, indicator species analyses, and differences in relative abundances can be found in Tables S10–S13.

By comparing differing land‐use sequences, we could further tease out the observed land‐use legacies on soil‐borne pathogens. Present‐day richness of fungal pathogens was higher in fields with a recent arable history, but this evened out toward the SNG baseline with increasing time since land‐use change (Figure 4A). Fungal pathogen richness moreover decreased with grassland afforestation. Notably, we found no corresponding pattern for potential pathogenic bacteria (Figure 4B).

Land‐use legacies were also evident in the composition of fungal pathogens, which generally differed between semi‐natural grasslands and sites with arable land‐use history (Permanova, *p* < 0.05 for all comparisons, except A>G 1800s; Figure 4C; Table S11). By contrast, the overall fungal community only differed between current and afforested grasslands (Table S11). Indicator species analyses (applied on pathogenic genera) revealed two fungal genera associated with sites with arable land‐use history which were additionally among the 10 pathogen genera with highest relative abundance: *Neoascochyta* and *Urocystis*. A corresponding genus (*Fusarium*) was associated with all current grasslands (Table S12). *Neoascochyta* and *Urocystis* reflected the observed legacy trends showing higher relative abundances in sites with arable land‐use history, which then decreased with time since conversion to grasslands (Figure 3D). These genera further decreased in relative abundances in afforested grasslands (ANCOM‐BC, *p* < 0.05 for all comparisons; Table S13). Conversely, *Cadophora* was singled out as an indicator of all except heavily afforested sites (i.e., G>F 1900s), and *Venturia* was indicative of current forests (G>F 1960s and G>F 1900s) but not grasslands. This latter genus also increased in relative abundance with time since grassland afforestation (Figure 4D). Notably, no bacterial potential pathogen genera were significantly associated with any LUS, and we therefore focused on fungal pathogen genera for subsequent analyses of legacy dilution pathways below.

### 3.4 Assessing the Factors Behind the Dilution of Land‐Use Legacies With Time

**FIGURE 5:**
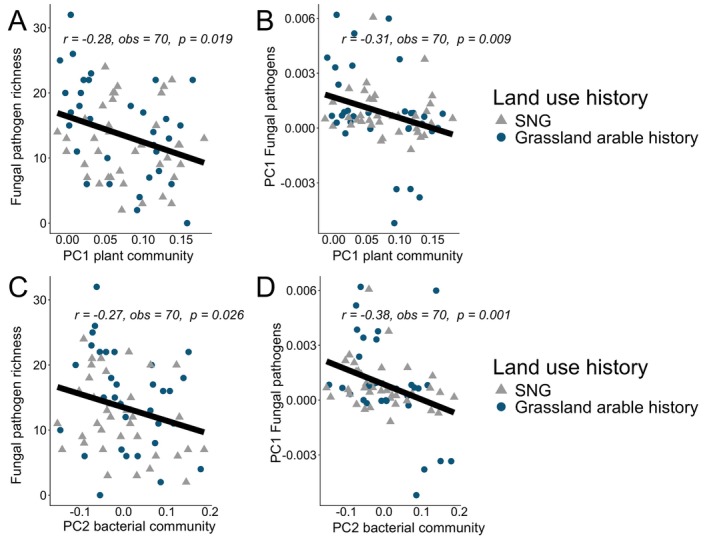
Links between fungal pathogen diversity and plant and bacterial community composition. Spearman correlations between fungal pathogen richness (A, C) and community composition (B, D) and plant and bacterial community composition. Community composition is represented through the first and second of principal component axes (i.e., PC1 and PC2) of the differing communities. Only currently managed grasslands (i.e., semi‐natural grasslands and grasslands with a history of arable land use) are shown.

The duration of legacy effects for soil‐borne fungal pathogens in sites with an arable land‐use history generally lasted up to a century after land‐use change only to attenuate and disappear over time. This was true for both richness, composition and relative abundances (Figure 4A–D). We next examined three putative pathways of legacy dilution: changes in soil abiotic properties (environmental filtering); links to plant communities through changes in plant richness and composition (above‐belowground linkages); and changing patterns in the association of microbial groups (microbial community dynamics). As bulk soil properties (Total C, Total N, C:N, bulk density, P, pH, P, K) remained similar across grasslands with and without a history of arable land use (Table S4), we ruled out environmental filtering through soil physicochemical properties as a main factor behind legacy dilution. Examining above‐belowground linkages, we found no significant correlations between plant and fungal pathogen richness (Table S14). However, the first principal component axis of plant community composition correlated significantly with fungal pathogen richness and composition across all grasslands with arable history and SNGs (Figure 5A,B). While this coincides with a shift from grasses to forbs observed in the PCA of plant functional types (Figure 2C), no aspect of fungal pathogen diversity correlated significantly with the diversity, cover, or ratio of forbs and grasses (Table S14).

**FIGURE 6:**
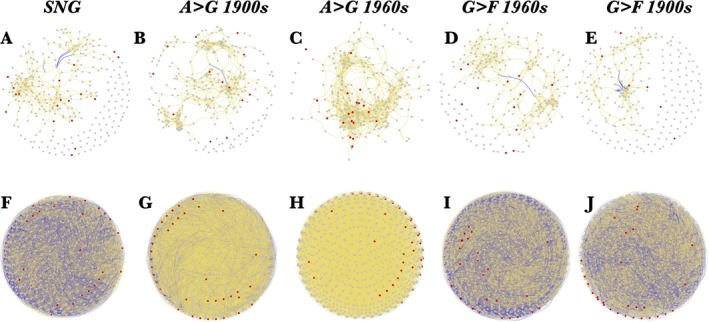
Reconfigurations of microbial networks with time since land‐use change. Co‐occurrence networks of fungal taxa (OTUs) across the differing land‐use sequences from grasslands with arable history (A, B), SNGs (C), and afforested grasslands (D, E). Corresponding networks for bacterial ASVs are displayed in (F–J). Colour of nodes indicates pathogenic (red) and other (grey) genera. Line colours indicate positive (golden) and negative (blue) correlation coefficients (Spearman's *r*) which include only links where *r* > 0.6 and *p* < 0.01.

Fungal pathogen richness and composition moreover correlated significantly with bacterial community composition (Principal component axis 2; Figure 5C,D), indicating co‐variation in microbial community dynamics. Examining co‐occurrence networks for each LUS (A>G 1800s was discarded due to insufficient sample size), we found that network size and clustering coefficients of fungal communities were highest in sites with recent arable history, only to then decrease with time since land‐use change toward SNG (Figure 6A–E; Table S15). Conversely, the average path length and network modularity, a measure of how much of the network is structured as cohesive subgroups of nodes (modules) in which the density of associations is higher within subgroups than among subgroups, were at their lowest in the same sites (Table S15). By contrast, bacterial networks remained roughly comparable across land‐use sequences (Figure 6F–J; Table S15).

## 4 Discussion

This study shows that persisting legacy effects from agricultural land use continue to shape the diversity and composition of present‐day microbial communities in semi‐natural grasslands, particularly soil‐borne plant pathogenic taxa. We also show that land‐use legacies can extend for at least 60–100 years after land use change but then decay over time and eventually disappear. This gives an indication of the temporal dynamics underpinning legacy effects and their dilution pathways.

### 4.1 Bacterial Legacies Last While Fungal Legacies Disappear Over Time

Land‐use legacies on plant communities have been extensively examined in the study areas investigated here and beyond (e.g., Gustavsson et al. 2007; Auffret et al. 2018), including persistent influence of land use more than 200 years ago on present‐day plant diversity. Seeing that our results broadly corroborate these findings (e.g., Figure 2A–C), we mainly focus on legacy responses of soil microbial communities which have not been examined previously. In line with our first hypothesis, we found that land‐use legacies shaped whole bacterial communities, but not fungi. Specifically, we found that land use in the 1900s explained significant variation in the overall richness and community composition of bacteria (Figures 3A and S3A). Intensified management tends to favour organisms with ‘fast’ life cycles like generalist arthropods, bacteria, ruderal plants and pathogenic fungi (Neyret et al. 2024), while disfavouring ‘slower’ organisms such as fungi or larger specialist soil animals. However, contrary to our hypothesis of stronger legacies for free‐living than for plant‐associated taxa, we found that both groups followed the overall community patterns for bacteria (Figures 3A and S3A), whereas only current land use affected plant‐associated fungi (Figure 3A). Fungal community assembly could be more independent of disturbances than bacteria (Powell et al. 2015), but it is well known that fungi are often affected by arable land use through, for example, disturbances and disruption of hyphal networks (Jasper et al. 1991; Schnoor et al. 2011). Our results could therefore indicate that, if disrupted by agricultural management, fungal communities reorganise within 40–60 years of grassland management, whereas bacteria and especially pathogen community disruptions remain imprinted in the soil as legacies for a longer time.

### 4.2 Soil‐Borne Plant Pathogens Are Key Indicators of Past Arable Land Use

Historical land use was of relatively higher importance than current land use in shaping the diversity of soil‐borne pathogens and potential pathogens (Figure 3B). Notably, fungal pathogen richness exhibited a legacy dilution pattern related to arable land use (Figure 4A), and additionally differed in their community composition between the SNG baseline and sites where conversion from arable to grassland occurred decades or a century ago (i.e., A>G 1960s and A>G 1900s; Figure 4C; Table S11). Higher pathogen loads in arable compared to grassland systems are well documented (French et al. 2017; Idbella and Bonanomi 2023; Labouyrie et al. 2023; Makiola et al. 2019) and generally relate to a build‐up of the soil‐borne pathogen pool in intensely managed farming systems (Morriën 2016) with more disturbances. Accordingly, Neyret et al. (2024) recently found that plant pathogens increase with intensification of grassland management. While these results broadly corroborate our hypothesis that the build‐up of soil‐borne pathogens with arable land use are visible for several decades after land‐use change, they also give an indication of the time it takes for these legacies to disappear.

By performing indicator taxa analyses and differential abundance testing on fungal pathogen genera across sites with differing land‐use histories (i.e., LUS), we could also examine genera‐specific dynamics underpinning decaying land‐use legacies more closely. Thus, *Neoascochyta* and *Urocystis*, both known to infect cereal crops (Aamot et al. 2020; Savchenko et al. 2017), were singled out as indicators of arable land‐use history and exhibited the characteristic dilution pattern with time since arable conversion to grassland (Figure 4D). By contrast, the more generalist *Fusarium* genus, known for their wide distribution across ecosystems and cosmopolitan host associations (Summerell et al. 2010), was an indicator of current grassland management and equally abundant across all managed grasslands independent of arable land‐use history (Table S13). Lastly, the genus *Venturia*, an important fruit crop pathogen known to associate with various *Prunus* hosts (González‐Domínguez et al. 2017) showed the opposite pattern of increased abundances with grassland afforestation and was consequently also an indicator of current forest land use. We note that in this case, plant ( *Prunus avium* ; Figure 2D) and pathogen (*Venturia*; Figure 4D) indicator species analyses coincide remarkably well, although we cannot ascertain the association to species level for the pathogens due to insufficient taxonomic resolution of the sequencing data.

### 4.3 Dilution of Legacy Effects Linked to Plant Community Composition and Microbial Association Structure

Controlled experiments over shorter time scales have shown that pathogen legacies weaken and disappear over time if other plant functional groups are introduced into the system (Hannula et al. 2021). Similarly, diversity‐driven dilution has been shown for crop overyielding within a year (Wang et al. 2023). Translated to the longer time periods of grassland after arable management in our study, it could be one of the potential pathways driving diminishing land‐use legacies over time in our systems. Other possible pathways relate to soil edaphic properties (van Agtmaal et al. 2017) and possibly slow changes in microbial interactions (de Boer et al. 2019). As we found no differences in the soil properties between grasslands with and without history of arable land use, environmental filtering through soil properties is unlikely to explain our results, although we acknowledge that differences may exist at the microhabitat level that are not captured by our soil sampling design (Albright et al. 2019). For instance, soil aggregate size and stability differ between arable land and grasslands (Spohn and Giani 2011), and these aggregates are known to shield entire microbial communities from biotic and abiotic stresses (Rillig et al. 2017). In contrast, we found tangible links between changing plant community properties (richness and the first axis from PCA analysis) and fungal pathogen richness and composition (Figure 5A,B, Table S14), suggesting that above‐belowground linkages play a role in the development of pathogen legacies after land‐use change.

We have previously found that both whole‐ and core microbial communities may exhibit threshold responses related to soil abiotic properties after land‐use change (Sveen et al. 2024). Our results here complicate this view, as alternative successional dynamics appear to be ingrained at the level of specific functional groups (i.e., fungal pathogens) which are inextricably linked to plant communities (Domínguez‐Begines et al. 2021; Kardol et al. 2007). Interestingly, the composition but not the richness of fungal pathogen composition strongly correlated with plant (Spearman *|r|* = 0.31, *p* = 0.009) and bacterial (Spearman *|r|* = 0.38, *p* < 0.001) community composition, suggesting that compositional co‐variation is stronger than, for example, links between plant and pathogen richness. Contrary to previous findings (Heinen et al. 2020), we found no indications that a change from grasses to forbs underpinned fungal pathogen legacy effects, but more precise profiling of, for example, rhizosphere and phyllosphere microbial communities in addition to the bulk soil communities examined here may be needed to determine links between plants and soil microbes.

We also analysed co‐occurrence network properties to examine potential links between changing microbial community dynamics after land‐use change and pathogens. When outside of the host plants, pathogens reside in the soil and are part of the overall soil microbial community (Termorshuizen and Jeger 2008) and may partake in interactions with other soil microbes. For instance, pathogens may become inhibited by secondary metabolites produced during interactions with other members of the soil microbial community (de Boer et al. 2019) or directly compete through the uptake of energy sources (Garbeva et al. 2011). The higher clustering coefficient of fungal networks in the sites most recently converted from arable to grasslands (A>G 1960s) may suggest that the persistence of pathogens in arable soils is upheld by the more disturbed and dynamic microbial communities found in arable soils compared to grasslands (Cornell et al. 2023), visible through a gradual reconfiguration of the microbial network occurring after land‐use conversion (Figure 6).

### 4.4 Limitations

Several limitations should be considered in the interpretation of this study's findings. The limitations inherent to space‐for‐time substitutions and inferring legacy effects based on baseline conditions have been mentioned above but merits further reflection. For instance, although land use categories are consistent across centuries, management factors within these categories have varied over time, often in the direction of increased intensification although this has been less pronounced in Sweden than elsewhere (Wretenberg et al. 2006). As the strength of legacy effects is typically proportional to the disturbance intensity of the system (Foster et al. 2003), stronger legacy signals could be expected from sites with a more recent history of arable land use than from the less intensive historical arable management practices, thus producing the pattern of gradually attenuating legacy effects over time (e.g., Figure 4A). Two things speak against this interpretation. Firstly, we would expect to see the same pattern reproduced across other groups of taxa than just pathogenic, something we did not observe. Secondly, land‐use legacies are generally visible in and mediated by modifications of soil properties (e.g., Dupouey et al. 2002), and these were indistinguishable between fields with and without agricultural land‐use history independent of time since land‐use change (Table S4). Crucially, this also means that our findings may be underestimating the size and duration of future legacies of the more intensive forms of arable farming today, including potentially higher levels of pathogens.

The time elapsed between soil sampling (June–September) and plant surveying (2015 vs. 2021) are additional sources of variation which have not been controlled for. However, plant communities in these sites have been shown to be stable across decadal timescales (Sara Cousins, *personal communication*). While grassland microbial communities can show strong seasonal dynamics (e.g., Hannula et al. 2019), yearly turnover rates are relatively low especially in soil compared to litter (Martinović et al. 2021). We therefore believe that these sources are likely to be minor. The caveats inherent to co‐occurrence analyses are many and well known (e.g., Blanchet et al. 2020), including confounding effects of shared environmental habitat. We note that results from co‐occurrence networks are indicative at best and should not be interpreted as evidence of interactions. Several other potential biotic pathways, all unmeasured here, including the transmission of pathogens through plant or insect vectors from the surrounding metacommunity, could also affect both the persistence and decay of the observed legacies (Martini et al. 2015).

Lastly, assigning pathogenicity status based on genus‐level taxonomic resolution for bacteria is challenging, because of the considerable inter‐ and intraspecific variation in lifestyle and pathogenicity (e.g., Lianou and Koutsoumanis 2013). This is likely reflected in our failure to capture legacy dilution patterns or indicator genera despite significant influence of historical (i.e., 20th century) land use on bacterial potential pathogens (Figure 3A). Sequencing technologies yielding metagenome‐assembled genomes (MAGs) combined with culturing techniques that allow for strain‐level differentiations could be a potential pathway forward to examine whether land‐use legacies work in similar ways for bacterial and fungal pathogens.

## 5 Conclusion

This study shows that legacy effects from historical land use continue to shape the ecosystems and microbial communities we observe today. Overall, bacteria show stronger legacies of past land use than fungi, but soil‐borne fungal pathogens emerged as a key group to follow with distinct signs of dependency on historical arable land use. Our findings suggest a temporal time frame of 50–100 years through which land‐use legacies and the processes leading to their eventual disappearance can be studied and understood, and we propose that specific genera of fungal pathogens can act as indicators of these legacies. However, for several reasons related to lower agricultural intensity in the past—up until the 1950s—it is highly likely that legacies of present‐day arable farming will be both stronger and more long‐lasting than found in our study.

## Author Contributions

T.R.S., E.G., S.C., M.V., J.B. and M.B. designed the study. T.R.S. did the fieldwork. T.R.S., F.B. and K.P. processed the samples. T.R.S. and I.J.M. analysed the data and performed the statistical analysis. T.R.S., J.B., M.V., M.B. and L.R. discussed, interpreted and wrote the manuscript, with input from all authors. All authors reviewed the manuscript.

## Funding

This work was supported by C.F. Lundströms Stiftelse, CF2023‐0019, Lars Hiertas Minne FO2021‐0302; Swedish University of Agricultural Sciences (early career grant), the Swedish Research Councils Formas (Grant 2020–00807), the Swedish Research Council (VR; Grant 2021–03724), Novo Nordisk Foundation (NNF24OC0089849), the Danish National Research Foundationa (DNRF183) and Stiftelsen Oscar och Lili Lamms Minne.

## Supporting information


**Data S1:** ele70332‐sup‐0001‐supinfo.docx.

## Data Availability

Raw sequences have been deposited at the NCBI database under the BioProjects PRJNA1238174. All data and codes needed to run analyses have been deposited at *Figshare*: https://figshare.com/projects/Microbial_land‐use_legacies/252773.
