# Supplementary material for: Soil‐Borne Pathogens Reflect Agricultural Land‐Use Legacies
Source: Ecol Lett. 2026 Feb 11;29(2):e70332. doi: 10.1111/ele.70332 (PMC12893405; doi:10.1111/ele.70332)
Supplement: Supplementary file 1 — Data S1: ele70332‐sup‐0001‐supinfo.docx. [file ELE-29-0-s001.docx]

**SUPPLEMENTARY MATERIALS**

Accompanying the article: ***Soil-borne pathogens reflect agricultural land-use legacies***

Tord Ranheim Sveen^1^*, Ida Junker Madsen^2^, Eva Gustavsson^3^, Sara Cousins^4^, Franz Buegger^5^, Karin Pritsch^5^, Laura Riggi^1,6^, Jan Bengtsson^1^, Maria Viketoft^1^, Mo Bahram^1,2,7^

^1^Department of Ecology, Swedish University of Agricultural Sciences, Ulls Väg 16, 75651 Uppsala, Sweden

^2^Department of Aroecology, Aarhus University, Forsøgsvej 1 4200, Slagelse, Denmark

^3^Department of Conservation, University of Gothenburg, Magasinsgatan 4, 54237 Mariestad, Sweden

^4^Department of Physical Geography, Stockholm University, Svante Ahrrenius Väg 8, Frescati Stockholm, Sweden

^5^Institute of Soil Ecology, Helmholtz Zentrum München, German Research Center for Environmental Health, Ingolstaedter Landstraße 1, D – 85764 Neuherberg, Germany

^6^Environmental Research, Wageningen University and Research, Wageningen, The Netherlands

^7^Institute of Ecology and Earth Sciences, University of Tartu, Tartu, Estonia

*****Corresponding author: [tord.ranheim.sveen@slu.se](mailto:tord.ranheim.sveen@slu.se)

**SUPPLEMENTARY METHODS**

*Site description*

The sites sampled for this study were derive from two regions in the South-West (Källtorp) and Central Sweden (Nynäs) and have been previously used to infer legacy effects of historical land use on plant communities and grassland specialist species (Gustavsson *et al.* 2007) and changes in land cover since the 1600s (Cousins 2001). In both regions, historical land-use maps were used to infer land-use changes between arable fields, semi-natural grasslands, and forests across four different time steps. The exact years of mapping vary between and within regions (see Cousins 2001 and Gustavsson et al. 2007 for exact mapping details) but are broadly consistent. In both regions, semi-natural grasslands (SNG), a practice forming the backbone of agriculture from the Bronze age and up until the mid-19^th^ century, (Eriksson & Cousins 2014) used to be the dominating land use, encompassing about 60 % of the landscape in the 1700s. The rest of the agricultural land was until the mid 1800s managed as agricultural fields using, compared to now, low-intensive agricultural methods, often as cereal crops interspersed with fallow periods every two- or three years, and fertilized by cattle manure, transporting nutrients from the permanent grasslands to arable fields. Remaining parts of the landscape were more or less forested, often used for livestock summer grazing (Eriksson & Cousins 2014). The proportion of SNGs has subsequently decreased to around 18 %, of which only 8 % are characterized by long-term continuity (Cousins 2001), mainly due to extensive abandonment and afforestation of previously grazed land during the 1900s.

*Soil processing and characterization*

We sampled soils from the centre coordinate of the site in four directions (1 m distance between subsamples) using a soil corer (diameter = 3 cm, depth = 10 cm), with 20 pooled subsamples making the final composite sample. All equipment was sterilized with 95 % ethanol between sites. A subset of each composite sample was frozen (-20 °C) within 24 hours of collection for molecular analyses, and the remaining sample was air-dried (< 40°C) before subsequent chemical analyses. Bulk density was measured from triplicate cores (depth = 10 cm, diameter = 5.5 cm). Approximately 20g of the soil was used to analyse pH (1:5 soil: water suspension), as well as available P and K using ICP-AES at Agrilab Uppsala, Sweden. Total C and N contents were determined on aliquots (1–20 mg) of air-dried soil using an Elemental Analyser (Euroa EA, Eurovector, Milano, Italy).

*DNA extraction and molecular analyses*

DNA was extracted from 200 mg of frozen soil samples using the PowerMax Soil DNA Isolation Mini kit (Qiagen GmbH, Hilden, Germany) following the manufacturer's instructions. Extraction occurred within 3 weeks of sampling. The extracted DNA was quality-checked based on the 260/280 and 260/230 nm wavelength ratios using a NanoDrop™ (Thermo Scientific, Massachusetts, USA) and stored at −20°C until sequencing. For the production of amplicons for sequencing, the universal prokaryote primers 515F and 806R were used to amplify the 16S V4 subregion of the rRNA gene (Walters *et al.* 2015). For fungi, the ITS4ngsUni-fITS7 primers targeting the second internal transcribed spacer region (ITS2) were used (Ihrmark *et al.* 2012; Tedersoo & Lindahl 2016). Samples were amplified using the following conditions in three replicate runs: For 16S, 95°C for 15 min, followed by 26 cycles of 95°C for 30 s, 50°C for 30 s and 72°C for 1 min with a final extension step at 72°C for 10 min. The 25 μL PCR mix consisted of 18 μL sterilised H_2_O, 5 μL 5 × HOT FIREPol Blend MasterMix 0.5 μL of each primer (20 μL), and 1 μL template DNA (final concentration of 400 nM). For ITS2 were amplified using the following conditions: 95°C for 10 min, followed by 30 cycles of 95°C for 30 s, 52°C for 30 s and 72°C for 45 s min with a final extension step at 72°C for 10 min. The amplicons from the replicates were pooled and purified using a purification kit containing agarose gel (FavourPrep Gel/PCR Purification mini Kit-300 Preps; Favourgen) and shipped for library preparation in the sequencing service facility of the University of Tartu (the Estonian Biocenter). DNA libraries were sequenced on two runs using an Illumina MiSeq platform (2 × 250 bp paired-end chemistry) at the Novogene Europe laboratory. Blanks containing ddH_2_O instead of DNA template were used as negative controls in the library preparation. The raw sequences have been deposited at NCBI under accession PRJNA1238174.

*Bioinformatics*

We used the LotuS2 version 2.22 (Özkurt *et al.* 2022) pipeline to quality-filter, demultiplex and process the filtered reads into operational taxonomic units (OTUs). Chimera detection and removal were done using Uchime (Edgar *et al.* 2011), and all singletons and sequences shorter than 100 bp were discarded. The clustering of sequences was done using a de-novo clustering algorithm in UPARSE (Edgar 2013) based on a 97% similarity threshold. Taxonomy was assigned against the SILVA database (Quast *et al.* 2013) (ver. 138.1) for prokaryotic sequences, and UNITE (ver. 10.0) (Abarenkov *et al.* 2024; Kõljalg *et al.* 2020) for fungal sequences. Mitochondral and chloroplast sequences were removed from the bacterial dataset, and sequences unassigned at phylum level were removed from bacterial and fungal datasets to avoid the inclusion of spurious OTUs. OTU matrices were rarefied to minimum sample depth to account for uneven sequencing depth, with an average of 66 230 and 20 741 sequences (± 23 571 and 11 087) for the bacterial and fungal datasets, respectively.

*Co-occurrence networks*

Networks were constructed, analyzed, and visualized using the *igraph* package in R (Csárdi *et al.* 2025). To reduce matrix sparsity, we applied an occupancy threshold, retaining only OTUs occurring at least 20 % of all sites within a given LUS (Weiss *et al.* 2016). A further significance threshold (Spearman’s *r* > 0.6 and *p* < 0.01) was set to include only significant co-occurrences. To optimize the balance between network accuracy and sample evenness, we randomly subsampled the LUS with the highest sample numbers (i.e. SNG and G>F 1900s) down to 30 samples. We kept other LUS sample sizes intact (Table 1) with the exception for the A>G 1800s treatment which was omitted due to insufficient sample depth (*n* = 5) (Kurtz *et al.* 2015). The final sample sizes did not correlate with any of the network properties, except for the ratio of positive and negative correlations for bacterial networks (Fig. S2B). Network modules were detected using the *cluster_fast_greedy* command, and modularity was calculated using the modularity command in *igraph*. We note that co-occurrence networks have been criticized for various reasons (e.g. Blanchet *et al.* 2020), but may still provide a valuable indication of community dynamics in the soil microbiome.

**SUPPLEMENTARY FIGURES**

**Figure S1: Rarefaction curves**

Rarefaction curves showing the relationship between taxa richness and sequencing depth for (A) fungal and (B) bacterial communities.

**Figure S2: Correlation between properties of microbial co-occurrence networks**

Spearman correlation between (**A**) fungal and (**B**) bacterial network properties including sample size. Cells with an X denotes non-significant (*p* < 0.05) correlation.

******

**Figure S3: Contribution of historical and current land use to present-day microbial community composition**

**A**) Explained variance (*R^2^*) of historical and current land use (LU) on present-day community composition of fungal and bacterial communities, and of their plant-associated and free-living taxa. In panel **B**) corresponding effects on pathogenic (fungi) and potentially pathogenic bacteiral taxa are shown. Asterisks (*) denote significant influence of land-use factor to present-day biodiversity based on permutational analyses (permanova) with the following significance levels: **p* < 0.05, ***p* < 0.01, ****p* < 0.001. Full test details for all groups can be found in Table S9.

**Figure S4: Land-use legacies in the present-day microbial richness**

Present-day richness of (**A**) bacteria and (**B**) fungi across sites classified into six differing land-use sequences (LUS) based on their respective land-use history and time since land-use change (Table 1). Asterisks (*) denote significant influence of land-use factor to present-day biodiversity based on GLMs with the following significance levels: **p* < 0.05, ***p* < 0.01, ****p* < 0.001. The dashed horizontal line indicates median pathogen richness of the baseline SNG sites.

**Figure S5: Contribution of historical and current land use to present-day microbial diversity using raw (i.e. non-rarefied) data**

**A**) The proportion (Explained variance, *R^2^*) of present-day microbial diversity attributed to current and historical land use (LU) across fungal and bacterial communities and their fractions of free-living and plant-associated taxa. In panel **B**) corresponding effects on beneficial and pathogenic taxa are shown. Asterisks (*) denote significant influence of land-use factor to present-day biodiversity based on GLMs with the following significance levels: **p* < 0.05, ***p* < 0.01, ****p* < 0.001.

**Figure S6: Legacies of arable land use in the present-day richness and composition of soil-borne pathogens using raw (i.e. non-rarefied) data**

Present-day richness of fungal (**A**) and bacterial (**B**) soil-borne pathogens across sites classified into six differing land-use sequences (LUS) based on their respective land-use history and time since land-use change (Table S2). Asterisks (*) denote significant influence of land-use factor to present-day biodiversity based on GLMs with the following significance levels: **p* < 0.05, ***p* < 0.01, ****p* < 0.001. The dashed horizontal line indicates median pathogen richness of the baseline SNG sites. **C**) PCoA of fungal pathogen community composition across the differing LUS. **D**) Relative abundances of five key fungal pathogenic genera of differing land-use histories. *Neoascochyta* and *Urocystis* are indicator genera for present-day grasslands with a history of arable land use. *Fusarium* is indicator genus of present-day grasslands, independent of arable land use history. *Venturia* indicates forests with a grassland history.

**SUPPLEMENTARY TABLES**

**Table S1**: Geographical location and environmental characteristics of the two study regions.

| **Study region** | ***Nynäs*** | ***Källtorp*** |
| --- | --- | --- |
| *Approximate Size (ha)* | 800 | 800 |
| *Geographical Midpoint* | 58°50’N, 17°24’E | 50°50’N, 12°91’E |
| *Mean annual precipitation (MAP)* | 600 mm | 600 mm |
| *Mean annual temperature (MAT)* | 7.0 °C | 6.5 °C |
| *Vegetation period (days)* | 184 | 195 |

**Table S2:** The study sites' historical and current land use (LU) are grouped into five land-use sequences (LUS) based on the change sequence between land uses across time.

| **LUS (replicates)** | *LU 18th c.* | *LU 19th c.* | *LU 20th c.* | *LU current*  *(from 2001)* |
| --- | --- | --- | --- | --- |
| *Semi-natural grassland* |  |  |  |  |
| SNG (38) | SNG | SNG | SNG | SNG |
| *Arable to grassland* |  |  |  |  |
| A>G 1800s (5) | Arable | SNG | SNG | SNG |
| A>G 1900s (11) | Arable | Arable | SNG | SNG |
| A>G 1960s (16) | Arable | Arable | Arable | SNG |
| *Grassland to forest* |  |  |  |  |
| G>F 1900s (42) | Grassland | Grassland | Abandoned | Forest |
| G>F 1960s (22) | Grassland | Grassland | Grassland | Forest |

**Table S3**: Sequencing depth of the 16S and ITS data, including the total reads after quality filtering and the distribution of OTUs and genera across whole communities and designated groups of plant-associated and pathogenic taxa.

| ***Dataset*** | ***Reads*** |  | ***Community*** | ***Taxa (OTUs)*** | ***Genera*** |
| --- | --- | --- | --- | --- | --- |
| *Soil bacteria* |  |  | *Bacteria* |  |  |
| sum | 6765955 |  | Whole community | 17705 | 1032 |
| mean | 50492 |  | Plant-associated | 1467 | 150 |
| sd | 19007 |  | Potential pathogenic | 465 | 29 |
| *Soil fungi* |  |  | *Fungi* |  |  |
| sum | 2086262 |  | Whole community | 4255 | 691 |
| mean | 15686 |  | Plant-associated | 697 | 187 |
| sd | 8872 |  | Pathogenic | 254 | 124 |

**Table S4**: Soil properties across the differing land-use sequences (LUS). Figures show mean and standard deviation, with superscript letters denoting significant differences (*p* < 0.05) based on pairwise Wilcoxon tests.

| **Soil property** | ***SNG (38)*** | ***A>G early (5)*** | ***A>G mid (11)*** | ***A>G late (16)*** | ***G>F early (42)*** | ***G>F late (22)*** |
| --- | --- | --- | --- | --- | --- | --- |
| *pH* | 5.26 (0.36) | 5.42 (0.31) | 5.19 (0.41) | 5.36 (0.62) | 5.09 (0.32) | 5.30 (0.54) |
| *Total C (%)* | 8.20 (4.85) | 7.05 (2.50) | 8.12 (3.17) | 9.31 (6.62) | 9.34 (3.83) | 8.23 (3.40) |
| *Total N (%)* | 0.61 (0.30) | 0.59 (0.21) | 0.64 (0.21) | 0.71 (0.48) | 0.55 (0.21) | 0.52 (0.24) |
| *C:N* | 13.4 (2.59)^a^ | 12.0 (0.40)^a^ | 12.8 (2.37)^a^ | 12.8 (1.52)^a^ | 17.3 (3.82)^b^ | 16.1 (3.91)^b^ |
| *BD^1^ (g cm^-3^)* | 1.01 (0.28)^a^ | 1.04 (0.36)^ab^ | 0.96 (0.17)^ab^ | 1.00 (0.37)^ab^ | 0.81 (0.25)^b^ | 1.00 (0.34)^ab^ |
| *K (mg kg^-1^)* | 23.3 (10.4) | 26.9 (13.5) | 31.6 (15.4) | 29.9 (13.4) | 19.9 (7.71) | 19.8 (5.61) |
| *P (mg kg^-1^)* | 6.98 (7.52) | 5.68 (3.85) | 8.66 (7.43) | 6.60 (4.77) | 4.53 (1.65) | 6.07 (6.22) |

^1^BD = Bulk density

**Table S5**: Parameter estimates from GLM assessing plant richness across sites divided into land-use sequences (LUS) based on their land-use history. Sites with a documented uninterrupted history of semi-natural grassland (SNG) manA>Gement were used as baseline for the comparisons.

| ***LUS*** | ***Estimate*** | ***Std. Error*** | ***t-value*** | ***Pr (>\|t\|)*** |
| --- | --- | --- | --- | --- |
| (Intercept) | 25.553 | 1.713 | 14.913 | 2.00 e-16*** |
| A>G 1960s | -5.615 | 3.148 | -1.784 | 0.076825 |
| A>G 1900s | -8.280 | 3.616 | -2.290 | 0.023685* |
| A>G 1800s | 3.847 | 5.025 | 0.766 | 0.445285 |
| G>F 1960s | -10.325 | 2.830 | -3.649 | 0.000382*** |
| G>F 1900s | -5.576 | 2.365 | -2.358 | 0.019884* |

**Table S6**: Results from multivariate permutational analyses of variance (perMANOVA) of plant communities across differing land-use sequences (LUS), based on Jaccard dissimilarity matrices and 9999 permutations. Benjamin-Hochberg corrections were applied to all raw *p*-values to adjust for multiple tests.

| ***Contrast*** | ***Df*** | ***SumsOfSqs*** | ***F*** | ***R2*** | ***p*** |
| --- | --- | --- | --- | --- | --- |
| SNG vs A>G 1960s | 1 | 0.54 | 1.35 | 0.02 | 0.138 |
| SNG vs G>F 1900s | 1 | 0.64 | 1.64 | 0.02 | 0.100 |
| SNG vs A>G 1900s | 1 | 0.43 | 1.08 | 0.02 | 0.394 |
| SNG vs G>F 1960s | 1 | 0.82 | 2.07 | 0.03 | 0.019* |
| SNG vs A>G 1800s | 1 | 0.35 | 0.90 | 0.02 | 0.646 |
| A>G 1960s vs G>F 1900s | 1 | 0.59 | 1.50 | 0.02 | 0.123 |
| A>G 1960s vs A>G 1900s | 1 | 0.38 | 0.92 | 0.03 | 0.631 |
| A>G 1960s vs G>F 1960s | 1 | 0.61 | 1.49 | 0.03 | 0.123 |
| A>G 1960s vs A>G 1800s | 1 | 0.40 | 1.00 | 0.05 | 0.510 |
| G>F 1900s vs A>G 1900s | 1 | 0.60 | 1.55 | 0.02 | 0.123 |
| G>F 1900s vs G>F 1960s | 1 | 0.49 | 1.25 | 0.01 | 0.232 |
| G>F 1900s vs A>G 1800s | 1 | 0.50 | 1.32 | 0.02 | 0.189 |
| A>G 1900s vs G>F 1960s | 1 | 0.72 | 1.80 | 0.05 | 0.091 |
| A>G 1900s vs A>G 1800s | 1 | 0.34 | 0.87 | 0.05 | 0.646 |
| G>F 1960s vs A>G 1800s | 1 | 0.63 | 1.60 | 0.06 | 0.12 |

**Table S7**: Plant species designated as indicators of differing land-use sequences (LUS) after indicator species analyses.

| ***LUS*** | ***Species*** | ***stat*** | ***p-value*** |
| --- | --- | --- | --- |
| A>G 1960s | *Epilobium angustifolium* | 0.433 | 0.0056** |
|  | *Cirsium palustre* | 0.385 | 0.0266* |
|  | *Alchemilla filicaulis* | 0.383 | 0.0298* |
|  | *Iris pseudacorus* | 0.383 | 0.0303* |
|  | *Galium palustre* | 0.368 | 0.0320* |
| A>G 1960s + A>G 1800s | *Prunella vulgaris* | 0.456 | 0.0297* |
| A>G 1960s + A>G 1900s | *Lathyrus pratensis* | 0.513 | 0.0322* |
| A>G 1960s + A>G 1900s + A>G 1800s | *Trifolium pratense* | 0.545 | 0.0049** |
|  | *Juncus effusus* | 0.483 | 0.0184* |
| A>G 1960s + SNG | *Trifolium medium* | 0.493 | 0.0226* |
| A>G 1900s + A>G 1800s + SNG | *Cerastium fontanum* | 0.608 | 0.0017** |
| A>G 1960s + A>G 1900s + A>G 1800s + SNG | *Trifolium repens* | 0.573 | 0.0130* |
|  | *Ranunculus acris* | 0.573 | 0.0289* |
| G>F 1960s + G>F 1900s | *Prunus avium* | 0.451 | 0.0458* |

**Table S8**: Results from Type II Anova tests of historical and current land use (LU) on present-day richness of microbial communities across different groups of free-living, plant-associated, and pathogenic taxa.

| ***Community*** | ***Landuse*** | χ^2^ | ***df*** | ***p-value*** |
| --- | --- | --- | --- | --- |
| ***Bacteria*** |  |  |  |  |
| Whole community | LU 1700s | 0.04 | 1 | 0.853 |
| Whole community | LU 1800s | 0.06 | 1 | 0.801 |
| Whole community | LU 1900s | 3.94 | 2 | 0.14 |
| Whole community | Current LU | 8.12 | 1 | 0.004** |
| Plant-associated | LU 1700s | 0.05 | 1 | 0.819 |
| Plant-associated | LU 1800s | 0 | 1 | 0.986 |
| Plant-associated | LU 1900s | 5.08 | 2 | 0.079. |
| Plant-associated | Current LU | 10.79 | 1 | 0.001** |
| Free-living | LU 1700s | 0.03 | 1 | 0.864 |
| Free-living | LU 1800s | 0.09 | 1 | 0.768 |
| Free-living | LU 1900s | 3.51 | 2 | 0.173 |
| Free-living | Current LU | 7.13 | 1 | 0.008** |
| ***Fungi*** |  |  |  |  |
| Whole community | LU 1700s | 0.01 | 1 | 0.932 |
| Whole community | LU 1800s | 0.6 | 1 | 0.439 |
| Whole community | LU 1900s | 4.67 | 2 | 0.097. |
| Whole community | Current LU | 1.22 | 1 | 0.269 |
| Plant-associated | LU 1700s | 0.66 | 1 | 0.417 |
| Plant-associated | LU 1800s | 0.14 | 1 | 0.711 |
| Plant-associated | LU 1900s | 0.36 | 2 | 0.835 |
| Plant-associated | Current LU | 7.05 | 1 | 0.008** |
| Free-living | LU 1700s | 0.17 | 1 | 0.678 |
| Free-living | LU 1800s | 0.41 | 1 | 0.523 |
| Free-living | LU 1900s | 5.97 | 2 | 0.051. |
| Free-living | Current LU | 3.56 | 1 | 0.059. |
| ***Pathogens*** |  |  |  |  |
| Bacterial potential pathogen | LU 1700s | 1.1 | 1 | 0.293 |
| Bacterial potential pathogen | LU 1800s | 2.67 | 1 | 0.102 |
| Bacterial potential pathogen | LU 1900s | 8.82 | 2 | 0.012* |
| Bacterial potential pathogen | Current LU | 2.08 | 1 | 0.149 |
| Fungal pathogen | LU 1700s | 0.16 | 1 | 0.687 |
| Fungal pathogen | LU 1800s | 0.04 | 1 | 0.848 |
| Fungal pathogen | LU 1900s | 12.9 | 2 | 0.002** |
| Fungal pathogen | Current LU | 0.73 | 1 | 0.392 |

**Table S9**: Results from permutational multivariate analysis of variance (perMANOVA) assessing the influence of historical and current land use (LU) on present-day community composition of microbes across different groups of free-living, plant-associated, and pathogenic taxa. perMANOVA models were based on Bray-Curtis distances with 9999 permutations

| ***Community*** | ***Landuse*** | ***df*** | ***SumOfSqs*** | ***R2*** | ***F*** | ***p-value*** |
| --- | --- | --- | --- | --- | --- | --- |
| ***Bacteria*** |  |  |  |  |  |  |
| Whole community | LU 1700s | 1 | 0.27 | 0.01 | 1.28 | 0.186 |
| Whole community | LU 1800s | 1 | 0.31 | 0.01 | 1.49 | 0.108 |
| Whole community | LU 1900s | 2 | 0.81 | 0.03 | 1.95 | 0.011* |
| Whole community | Current LU | 1 | 0.64 | 0.02 | 3.12 | 0.002** |
| Plant-associated | LU 1700s | 1 | 0.17 | 0.01 | 1.02 | 0.394 |
| Plant-associated | LU 1800s | 1 | 0.16 | 0.01 | 0.92 | 0.491 |
| Plant-associated | LU 1900s | 2 | 0.62 | 0.02 | 1.79 | 0.016* |
| Plant-associated | Current LU | 1 | 0.75 | 0.03 | 4.39 | 0.000*** |
| Free-living | LU 1700s | 1 | 0.27 | 0.01 | 1.29 | 0.18 |
| Free-living | LU 1800s | 1 | 0.32 | 0.01 | 1.53 | 0.101 |
| Free-living | LU 1900s | 2 | 0.83 | 0.03 | 1.96 | 0.011* |
| Free-living | Current LU | 1 | 0.62 | 0.02 | 2.95 | 0.004** |
| ***Fungi*** |  |  |  |  |  |  |
| Whole community | LU 1700s | 1 | 0.35 | 0.01 | 0.94 | 0.559 |
| Whole community | LU 1800s | 1 | 0.38 | 0.01 | 1.02 | 0.381 |
| Whole community | LU 1900s | 2 | 0.82 | 0.02 | 1.11 | 0.188 |
| Whole community | Current LU | 1 | 1.01 | 0.02 | 2.7 | 0.000*** |
| Plant-associated | LU 1700s | 1 | 0.37 | 0.01 | 0.85 | 0.788 |
| Plant-associated | LU 1800s | 1 | 0.43 | 0.01 | 0.98 | 0.478 |
| Plant-associated | LU 1900s | 2 | 0.85 | 0.01 | 0.96 | 0.566 |
| Plant-associated | Current LU | 1 | 0.86 | 0.01 | 1.95 | 0.001*** |
| Free-living | LU 1700s | 1 | 0.35 | 0.01 | 1 | 0.401 |
| Free-living | LU 1800s | 1 | 0.39 | 0.01 | 1.12 | 0.225 |
| Free-living | LU 1900s | 2 | 0.87 | 0.02 | 1.24 | 0.074. |
| Free-living | Current LU | 1 | 1.05 | 0.02 | 3.01 | 0*** |
| ***Pathogens*** |  |  |  |  |  |  |
| Bacterial potential pathogen | LU 1700s | 1 | 0.2 | 0.01 | 0.98 | 0.419 |
| Bacterial potential pathogen | LU 1800s | 1 | 0.14 | 0 | 0.7 | 0.761 |
| Bacterial potential pathogen | LU 1900s | 2 | 0.76 | 0.03 | 1.87 | 0.014* |
| Bacterial potential pathogen | Current LU | 1 | 0.81 | 0.03 | 4.02 | 0.000*** |
| Fungal pathogen | LU 1700s | 1 | 0.41 | 0.01 | 0.97 | 0.524 |
| Fungal pathogen | LU 1800s | 1 | 0.6 | 0.01 | 1.41 | 0.044* |
| Fungal pathogen | LU 1900s | 2 | 0.82 | 0.01 | 0.97 | 0.553 |
| Fungal pathogen | Current LU | 1 | 1.04 | 0.02 | 2.45 | 0.000*** |

**Table S10**: Parameter estimates from GLM assessing microbial richness across sites divided into land-use sequences (LUS) based on their land-use history. Sites with a documented uninterrupted history of semi-natural grassland (SNG) management were used as baseline for the comparisons.

| ***Community*** | ***Estimate*** | ***se*** | ***t-value*** | ***Pr(>\|t\|)*** |
| --- | --- | --- | --- | --- |
| ***Bacterial richness*** |  |  |  |  |
| *(Intercept)* | 1571 | 56.4 | 27.8 | <2e-16*** |
| *A>G 1960s* | 4.72 | 103.61 | 0.046 | 0.96373 |
| *A>G 1900s* | 14.79 | 119.03 | 0.124 | 0.90128 |
| *A>G 1800s* | -207 | 165 | -1.26 | 0.211 |
| *G>F 1960s* | 246.20 | 93.14 | 2.643 | 0.009** |
| *G>F 1900s* | 72.68 | 77.83 | 0.934 | 0.35217 |
| ***Plant-associated bacteria*** |  |  |  |  |
| *(Intercept)* | 163.658 | 9.271 | 17.653 | <2e-16*** |
| *A>G 1960s* | 16.780 | 17.031 | 0.985 | 0.32637 |
| *A>G 1900s* | 10.615 | 19.566 | 0.543 | 0.58841 |
| *A>G 1800s* | -21.658 | 27.187 | -0.797 | 0.42714 |
| *G>F 1960s* | 17.675 | 12.795 | 1.381 | 0.16954 |
| *G>F 1900s* | 48.251 | 15.310 | 3.152 | 0.002** |
| ***Free-living bacteria*** |  |  |  |  |
| *(Intercept)* | 1408.184 | 48.808 | 28.851 | <2e-16*** |
| *A>G 1960s* | -12.059 | 89.667 | -0.134 | 0.8932 |
| *A>G 1900s* | 4.179 | 103.014 | 0.041 | 0.9677 |
| *A>G 1800s* | -186.184 | 143.134 | -1.301 | 0.1957 |
| *G>F 1960s* | 197.952 | 80.604 | 2.456 | 0.0154* |
| *G>F 1900s* | 55.006 | 67.362 | 0.817 | 0.4157 |
| ***Fungal richness*** |  |  |  |  |
| *(Intercept)* | 218.895 | 6.959 | 31.455 | <2e-16*** |
| *A>G 1960s* | 23.793 | 12.785 | 1.861 | 0.06505 |
| *A>G 1900s* | 15.560 | 14.688 | 1.059 | 0.29144 |
| *A>G 1800s* | 4.905 | 20.408 | 0.240 | 0.81044 |
| *G>F 1960s* | -13.486 | 11.493 | -1.173 | 0.24282 |
| *G>F 1900s* | -32.431 | 9.660 | -3.357 | 0.001** |
| ***Plant-associated fungi*** |  |  |  |  |
| *(Intercept)* | 27.4211 | 1.9873 | 13.798 | <2e-16*** |
| *A>G 1960s* | -0.3586 | 3.6510 | -0.098 | 0.9219 |
| *A>G 1900s* | -1.8756 | 4.1944 | -0.447 | 0.6555 |
| *A>G 1800s* | -6.8211 | 5.8280 | -1.170 | 0.2440 |
| *G>F 1960s* | 8.0789 | 3.2820 | 2.462 | 0.015* |
| *G>F 1900s* | 6.5058 | 2.7586 | 2.358 | 0.012* |
| ***Free-living fungi*** |  |  |  |  |
| *(Intercept)* | 200.237 | 6.724 | 29.781 | <2e-16*** |
| *A>G 1960s* | 27.263 | 12.352 | 2.207 | 0.029* |
| *A>G 1900s* | 17.854 | 14.191 | 1.258 | 0.2107 |
| *A>G 1800s* | 11.363 | 19.718 | 0.576 | 0.5654 |
| *G>F 1960s* | -21.055 | 11.104 | -1.896 | 0.0602 |
| *G>F 1900s* | -41.773 | 9.333 | -4.476 | 1.67e-05*** |
| ***Bacterial potential pathogen*** |  |  |  |  |
| *(Intercept)* | 62.579 | 2.435 | 25.695 | <2e-16*** |
| *A>G 1960s* | 2.109 | 4.474 | 0.471 | 0.6383 |
| *A>G 1900s* | 5.967 | 5.140 | 1.161 | 0.2479 |
| *A>G 1800s* | -11.579 | 7.142 | -1.621 | 0.1074 |
| *G>F 1960s* | 5.921 | 4.022 | 1.472 | 0.1434 |
| *G>F 1900s* | -5.817 | 3.361 | -1.731 | 0.0859 |
| ***Fungal pathogen*** |  |  |  |  |
| *(Intercept)* | 12.2105 | 0.9368 | 13.034 | <2e-16*** |
| *A>G 1960s* | 4.7270 | 1.7211 | 2.746 | 0.007** |
| *A>G 1900s* | 1.3349 | 1.9773 | 0.675 | 0.5008 |
| *A>G 1800s* | -1.0105 | 2.7474 | -0.368 | 0.7136 |
| *G>F 1960s* | 1.3804 | 1.5472 | 0.892 | 0.3740 |
| *G>F 1900s* | -3.1130 | 1.3004 | -2.394 | 0.018* |

**Table S11**: Results from multivariate permutational analyses of variance (perMANOVA) of microbial communities across differing land-use sequences (LUS), based on Bray-Curtis dissimilarity matrices and 9999 permutations. Benjamin-Hochberg corrections were applied to all raw *p*-values to adjust for multiple tests.

| ***Community*** | ***Contrast*** | ***df*** | ***R2*** | ***F*** | ***p-value*** |
| --- | --- | --- | --- | --- | --- |
| ***Bacteria*** |  |  |  |  |  |
| Whole community | SNG vs AG 1960s | 1 | 0.03 | 1.73 | 0.093. |
| Whole community | SNG vs GF 1900s | 1 | 0.09 | 7.37 | 0.006** |
| Whole community | SNG vs AG 1900s | 1 | 0.03 | 1.2 | 0.221 |
| Whole community | SNG vs GF 1960s | 1 | 0.05 | 3.02 | 0.006** |
| Whole community | SNG vs AG 1800s | 1 | 0.02 | 1 | 0.388 |
| Whole community | AG 1960s vs GF 1900s | 1 | 0.09 | 5.76 | 0.006** |
| Whole community | AG 1960s vs AG 1900s | 1 | 0.06 | 1.56 | 0.149 |
| Whole community | AG 1960s vs GF 1960s | 1 | 0.05 | 1.96 | 0.075. |
| Whole community | AG 1960s vs AG 1800s | 1 | 0.07 | 1.33 | 0.202 |
| Whole community | GF 1900s vs AG 1900s | 1 | 0.06 | 3.57 | 0.006** |
| Whole community | GF 1900s vs GF 1960s | 1 | 0.04 | 2.85 | 0.007** |
| Whole community | GF 1900s vs AG 1800s | 1 | 0.07 | 3.25 | 0.006** |
| Whole community | AG 1900s vs GF 1960s | 1 | 0.07 | 2.44 | 0.032* |
| Whole community | AG 1900s vs AG 1800s | 1 | 0.09 | 1.44 | 0.176 |
| Whole community | GF 1960s vs AG 1800s | 1 | 0.07 | 2.03 | 0.039* |
| Plant-associated | SNG vs AG 1960s | 1 | 0.03 | 1.64 | 0.09. |
| Plant-associated | SNG vs GF 1900s | 1 | 0.09 | 7.58 | 0.003** |
| Plant-associated | SNG vs AG 1900s | 1 | 0.03 | 1.38 | 0.159 |
| Plant-associated | SNG vs GF 1960s | 1 | 0.07 | 4.37 | 0.003** |
| Plant-associated | SNG vs AG 1800s | 1 | 0.01 | 0.59 | 0.92 |
| Plant-associated | AG 1960s vs GF 1900s | 1 | 0.1 | 6.15 | 0.003** |
| Plant-associated | AG 1960s vs AG 1900s | 1 | 0.07 | 1.79 | 0.066. |
| Plant-associated | AG 1960s vs GF 1960s | 1 | 0.07 | 2.72 | 0.01** |
| Plant-associated | AG 1960s vs AG 1800s | 1 | 0.04 | 0.81 | 0.66 |
| Plant-associated | GF 1900s vs AG 1900s | 1 | 0.1 | 5.39 | 0.003** |
| Plant-associated | GF 1900s vs GF 1960s | 1 | 0.04 | 2.22 | 0.034* |
| Plant-associated | GF 1900s vs AG 1800s | 1 | 0.05 | 2.47 | 0.032* |
| Plant-associated | AG 1900s vs GF 1960s | 1 | 0.12 | 4.25 | 0.003** |
| Plant-associated | AG 1900s vs AG 1800s | 1 | 0.06 | 0.94 | 0.578 |
| Plant-associated | GF 1960s vs AG 1800s | 1 | 0.07 | 2.03 | 0.05* |
| Free-living | SNG vs AG 1960s | 1 | 0.03 | 1.73 | 0.087. |
| Free-living | SNG vs GF 1900s | 1 | 0.09 | 7.32 | 0.005** |
| Free-living | SNG vs AG 1900s | 1 | 0.02 | 1.17 | 0.248 |
| Free-living | SNG vs GF 1960s | 1 | 0.05 | 2.85 | 0.009** |
| Free-living | SNG vs AG 1800s | 1 | 0.02 | 1.02 | 0.372 |
| Free-living | AG 1960s vs GF 1900s | 1 | 0.09 | 5.72 | 0.005** |
| Free-living | AG 1960s vs AG 1900s | 1 | 0.06 | 1.53 | 0.16 |
| Free-living | AG 1960s vs GF 1960s | 1 | 0.05 | 1.89 | 0.06. |
| Free-living | AG 1960s vs AG 1800s | 1 | 0.07 | 1.35 | 0.19 |
| Free-living | GF 1900s vs AG 1900s | 1 | 0.06 | 3.35 | 0.009** |
| Free-living | GF 1900s vs GF 1960s | 1 | 0.04 | 2.9 | 0.022* |
| Free-living | GF 1900s vs AG 1800s | 1 | 0.07 | 3.31 | 0.005** |
| Free-living | AG 1900s vs GF 1960s | 1 | 0.07 | 2.23 | 0.054. |
| Free-living | AG 1900s vs AG 1800s | 1 | 0.1 | 1.47 | 0.165 |
| Free-living | GF 1960s vs AG 1800s | 1 | 0.07 | 1.99 | 0.054. |
| Potential pathogen | SNG vs AG 1960s | 0.03 | 1.68 | 0.117 | 0.117 |
| Potential pathogen | SNG vs GF 1900s | 0.09 | 7.81 | 0.005 | 0.005** |
| Potential pathogen | SNG vs AG 1900s | 0.02 | 1.08 | 0.414 | 0.414 |
| Potential pathogen | SNG vs GF 1960s | 0.06 | 3.83 | 0.007 | 0.007** |
| Potential pathogen | SNG vs AG 1800s | 0.01 | 0.6 | 0.905 | 0.905 |
| Potential pathogen | AG 1960s vs GF 1900s | 0.11 | 6.69 | 0.005 | 0.005** |
| Potential pathogen | AG 1960s vs AG 1900s | 0.06 | 1.48 | 0.142 | 0.142 |
| Potential pathogen | AG 1960s vs GF 1960s | 0.07 | 2.58 | 0.024 | 0.024* |
| Potential pathogen | AG 1960s vs AG 1800s | 0.05 | 0.95 | 0.518 | 0.518 |
| Potential pathogen | GF 1900s vs AG 1900s | 0.07 | 4.03 | 0.005 | 0.005** |
| Potential pathogen | GF 1900s vs GF 1960s | 0.04 | 2.87 | 0.02 | 0.02* |
| Potential pathogen | GF 1900s vs AG 1800s | 0.05 | 2.33 | 0.045 | 0.045* |
| Potential pathogen | AG 1900s vs GF 1960s | 0.09 | 3.04 | 0.009 | 0.009** |
| Potential pathogen | AG 1900s vs AG 1800s | 0.04 | 0.64 | 0.905 | 0.905 |
| Potential pathogen | GF 1960s vs AG 1800s | 0.07 | 1.96 | 0.06 | 0.06. |
| ***Fungi*** |  |  |  |  |  |
| Whole commmunity | SNG vs AG 1960s | 0.02 | 1.11 | 0.314 | 0.314 |
| Whole commmunity | SNG vs GF 1900s | 0.07 | 5.55 | 0.002 | 0.002** |
| Whole commmunity | SNG vs AG 1900s | 0.02 | 1.13 | 0.314 | 0.314 |
| Whole commmunity | SNG vs GF 1960s | 0.04 | 2.36 | 0.002 | 0.002** |
| Whole commmunity | SNG vs AG 1800s | 0.02 | 0.97 | 0.528 | 0.528 |
| Whole commmunity | AG 1960s vs GF 1900s | 0.08 | 4.82 | 0.002 | 0.002** |
| Whole commmunity | AG 1960s vs AG 1900s | 0.04 | 1.09 | 0.376 | 0.376 |
| Whole commmunity | AG 1960s vs GF 1960s | 0.06 | 2.31 | 0.002 | 0.002** |
| Whole commmunity | AG 1960s vs AG 1800s | 0.05 | 0.96 | 0.528 | 0.528 |
| Whole commmunity | GF 1900s vs AG 1900s | 0.06 | 3.13 | 0.002 | 0.002** |
| Whole commmunity | GF 1900s vs GF 1960s | 0.02 | 1.43 | 0.028 | 0.028* |
| Whole commmunity | GF 1900s vs AG 1800s | 0.05 | 2.51 | 0.002 | 0.002** |
| Whole commmunity | AG 1900s vs GF 1960s | 0.06 | 1.87 | 0.002 | 0.002** |
| Whole commmunity | AG 1900s vs AG 1800s | 0.07 | 1.07 | 0.376 | 0.376 |
| Whole commmunity | GF 1960s vs AG 1800s | 0.06 | 1.71 | 0.007 | 0.007** |
| Plant-associated | SNG vs AG 1960s | 0.02 | 1.27 | 0.182 | 0.182 |
| Plant-associated | SNG vs GF 1900s | 0.05 | 3.67 | 0.005 | 0.005** |
| Plant-associated | SNG vs AG 1900s | 0.02 | 0.95 | 0.632 | 0.632 |
| Plant-associated | SNG vs GF 1960s | 0.03 | 1.69 | 0.012 | 0.012* |
| Plant-associated | SNG vs AG 1800s | 0.02 | 0.82 | 0.859 | 0.859 |
| Plant-associated | AG 1960s vs GF 1900s | 0.07 | 4.34 | 0.005 | 0.005** |
| Plant-associated | AG 1960s vs AG 1900s | 0.04 | 1.13 | 0.322 | 0.322 |
| Plant-associated | AG 1960s vs GF 1960s | 0.06 | 2.47 | 0.005 | 0.005** |
| Plant-associated | AG 1960s vs AG 1800s | 0.05 | 1.03 | 0.483 | 0.483 |
| Plant-associated | GF 1900s vs AG 1900s | 0.04 | 2.12 | 0.007 | 0.007** |
| Plant-associated | GF 1900s vs GF 1960s | 0.02 | 1.07 | 0.434 | 0.434 |
| Plant-associated | GF 1900s vs AG 1800s | 0.04 | 1.78 | 0.015 | 0.015* |
| Plant-associated | AG 1900s vs GF 1960s | 0.05 | 1.56 | 0.013 | 0.013* |
| Plant-associated | AG 1900s vs AG 1800s | 0.06 | 0.88 | 0.718 | 0.718 |
| Plant-associated | GF 1960s vs AG 1800s | 0.05 | 1.25 | 0.133 | 0.133 |
| Free-living | SNG vs AG 1960s | 0.02 | 1.1 | 0.312 | 0.312 |
| Free-living | SNG vs GF 1900s | 0.08 | 6.58 | 0.003 | 0.003** |
| Free-living | SNG vs AG 1900s | 0.03 | 1.2 | 0.217 | 0.217 |
| Free-living | SNG vs GF 1960s | 0.04 | 2.57 | 0.004 | 0.004** |
| Free-living | SNG vs AG 1800s | 0.02 | 1.01 | 0.439 | 0.439 |
| Free-living | AG 1960s vs GF 1900s | 0.09 | 5.74 | 0.003 | 0.003** |
| Free-living | AG 1960s vs AG 1900s | 0.04 | 1.1 | 0.317 | 0.317 |
| Free-living | AG 1960s vs GF 1960s | 0.06 | 2.48 | 0.003 | 0.003** |
| Free-living | AG 1960s vs AG 1800s | 0.05 | 0.96 | 0.522 | 0.522 |
| Free-living | GF 1900s vs AG 1900s | 0.07 | 3.82 | 0.003 | 0.003** |
| Free-living | GF 1900s vs GF 1960s | 0.03 | 1.7 | 0.012 | 0.012* |
| Free-living | GF 1900s vs AG 1800s | 0.06 | 2.98 | 0.003 | 0.003** |
| Free-living | AG 1900s vs GF 1960s | 0.06 | 2.06 | 0.004 | 0.004** |
| Free-living | AG 1900s vs AG 1800s | 0.07 | 1.13 | 0.312 | 0.312 |
| Free-living | GF 1960s vs AG 1800s | 0.07 | 1.88 | 0.004 | 0.004** |
| Pathogen | SNG vs AG 1960s | 0.02 | 1.26 | 0.18 | 0.18 |
| Pathogen | SNG vs GF 1900s | 0.04 | 3.4 | 0.003 | 0.003** |
| Pathogen | SNG vs AG 1900s | 0.03 | 1.43 | 0.075 | 0.075. |
| Pathogen | SNG vs GF 1960s | 0.04 | 2.24 | 0.003 | 0.003** |
| Pathogen | SNG vs AG 1800s | 0.02 | 0.89 | 0.66 | 0.66 |
| Pathogen | AG 1960s vs GF 1900s | 0.06 | 3.42 | 0.003 | 0.003** |
| Pathogen | AG 1960s vs AG 1900s | 0.03 | 0.87 | 0.66 | 0.66 |
| Pathogen | AG 1960s vs GF 1960s | 0.06 | 2.43 | 0.003 | 0.003** |
| Pathogen | AG 1960s vs AG 1800s | 0.05 | 1.01 | 0.467 | 0.467 |
| Pathogen | GF 1900s vs AG 1900s | 0.04 | 2.19 | 0.005 | 0.005** |
| Pathogen | GF 1900s vs GF 1960s | 0.02 | 1.25 | 0.2 | 0.2 |
| Pathogen | GF 1900s vs AG 1800s | 0.03 | 1.41 | 0.077 | 0.077. |
| Pathogen | AG 1900s vs GF 1960s | 0.07 | 2.32 | 0.003 | 0.003** |
| Pathogen | AG 1900s vs AG 1800s | 0.08 | 1.18 | 0.315 | 0.315 |
| Pathogen | GF 1960s vs AG 1800s | 0.04 | 1.08 | 0.413 | 0.413 |

**Table S12**: Fungal pathogenic genera singled out as indicators of differing land-use sequences (LUS) after indicator species analyses.

| ***Genus*** | ***Relative abundance (%)*** | ***Indicator LUS*** | ***Stat*** | ***p*** |
| --- | --- | --- | --- | --- |
| Neoascochyta | 3.90 | A>G 1800s + A>G 1900s + A>G 1960s | 0.791 | 0.004** |
| Urocystis | 3.79 | A>G 1800s + A>G 1900s + A>G 1960s | 0.779 | 0.002** |
| Fusarium | 7.40 | A>G 1800s + A>G 1900s + A>G 1960s + SNG | 0.844 | < 0.001*** |
| Cadophora | 4.07 | A>G 1800s + A>G 1900s + A>G 1960s + SNG + G>F 1960s | 0.713 | 0.011* |
| Venturia | 10.0 | G>F 1960s + G>F 1900s | 0.827 | 0.003** |

**Table S13**: Results from differential abundance analyses of indicator fungal pathogen genera between sites across differing land-use sequences (LUS). Significant values (*p* < 0.05) indicate that the genera differ in their relative abundances between LUS based on analyses of composition with bias correction (ANCOM-BC).

| ***Genus*** | ***Contrast*** | ***lfc*** | ***se*** | ***W*** | ***p-value*** | ***q-value*** |
| --- | --- | --- | --- | --- | --- | --- |
| Diaporthe | SNG vs A>G 1960s | 0.443 | 0.312 | 1.421 | 0.155 | 0.522 |
| Diaporthe | SNG vs A>G 1900s | -0.092 | 0.316 | -0.291 | 0.771 | 0.881 |
| Diaporthe | SNG vs A>G 1800s | 0.498 | 0.44 | 1.132 | 0.258 | 0.718 |
| Diaporthe | SNG vs G>F 1900s | -0.595 | 0.17 | -3.493 | <0.001*** | 0.002 |
| Diaporthe | SNG vs G>F 1960s | -0.355 | 0.243 | -1.461 | 0.144 | 0.256 |
| Diaporthe | A>G 1800s vs A>G 1900s | -0.577 | 0.501 | -1.153 | 0.249 | 0.606 |
| Diaporthe | A>G 1800s vs A>G 1960s | 0.079 | 0.498 | 0.158 | 0.874 | 0.897 |
| Diaporthe | A>G 1800s vs G>F 1900s | -1.003 | 0.424 | -2.365 | 0.018* | 0.088 |
| Diaporthe | A>G 1800s vs G>F 1960s | -0.633 | 0.458 | -1.382 | 0.167 | 0.531 |
| Diaporthe | A>G 1900s vs A>G 1960s | 0.581 | 0.392 | 1.481 | 0.139 | 0.491 |
| Diaporthe | A>G 1900s vs G>F 1900s | -0.447 | 0.293 | -1.528 | 0.127 | 0.411 |
| Diaporthe | A>G 1900s vs G>F 1960s | -0.143 | 0.34 | -0.421 | 0.673 | 0.906 |
| Diaporthe | A>G 1960s vs G>F 1900s | -1.166 | 0.289 | -4.038 | <0.001*** | 0 |
| Diaporthe | A>G 1960s vs G>F 1960s | -0.713 | 0.337 | -2.118 | 0.034* | 0.148 |
| Diaporthe | G>F 1960s vs G>F 1900s | -0.236 | 0.213 | -1.11 | 0.267 | 0.694 |
| Fusarium | SNG vs A>G 1960s | 0.445 | 0.305 | 1.457 | 0.145 | 0.522 |
| Fusarium | SNG vs A>G 1900s | -0.305 | 0.311 | -0.981 | 0.327 | 0.852 |
| Fusarium | SNG vs A>G 1800s | 1.089 | 0.667 | 1.631 | 0.103 | 0.56 |
| Fusarium | SNG vs G>F 1900s | -1.091 | 0.232 | -4.705 | <0.001*** | 0 |
| Fusarium | SNG vs G>F 1960s | -0.522 | 0.294 | -1.778 | 0.075. | 0.164 |
| Fusarium | A>G 1800s vs A>G 1900s | -1.381 | 0.684 | -2.019 | 0.043* | 0.312 |
| Fusarium | A>G 1800s vs A>G 1960s | -0.51 | 0.681 | -0.749 | 0.454 | 0.768 |
| Fusarium | A>G 1800s vs G>F 1900s | -2.09 | 0.652 | -3.206 | 0.001*** | 0.01 |
| Fusarium | A>G 1800s vs G>F 1960s | -1.391 | 0.676 | -2.056 | 0.04* | 0.31 |
| Fusarium | A>G 1900s vs A>G 1960s | 0.796 | 0.34 | 2.341 | 0.019* | 0.25 |
| Fusarium | A>G 1900s vs G>F 1900s | -0.731 | 0.276 | -2.645 | 0.008** | 0.08 |
| Fusarium | A>G 1900s vs G>F 1960s | -0.097 | 0.33 | -0.295 | 0.768 | 0.972 |
| Fusarium | A>G 1960s vs G>F 1900s | -1.664 | 0.27 | -6.172 | <0.001*** | 0 |
| Fusarium | A>G 1960s vs G>F 1960s | -0.882 | 0.324 | -2.721 | 0.007** | 0.062 |
| Fusarium | G>F 1960s vs G>F 1900s | -0.566 | 0.256 | -2.206 | 0.027* | 0.213 |
| Neoascochyta | SNG vs A>G 1960s | 0.657 | 0.423 | 1.553 | 0.012* | 0.522 |
| Neoascochyta | SNG vs A>G 1900s | 1.09 | 0.392 | 2.78 | 0.005** | 0.106 |
| Neoascochyta | SNG vs A>G 1800s | 0.111 | 0.458 | 0.243 | 0.808 | 0.948 |
| Neoascochyta | SNG vs G>F 1900s | -0.532 | 0.166 | -3.212 | 0.001*** | 0.005 |
| Neoascochyta | SNG vs G>F 1960s | -0.509 | 0.201 | -2.529 | 0.011* | 0.042 |
| Neoascochyta | A>G 1800s vs A>G 1900s | 0.991 | 0.566 | 1.751 | 0.08. | 0.312 |
| Neoascochyta | A>G 1800s vs A>G 1960s | 0.679 | 0.588 | 1.156 | 0.248 | 0.585 |
| Neoascochyta | A>G 1800s vs G>F 1900s | -0.553 | 0.441 | -1.256 | 0.209 | 0.389 |
| Neoascochyta | A>G 1800s vs G>F 1960s | -0.4 | 0.455 | -0.879 | 0.379 | 0.617 |
| Neoascochyta | A>G 1900s vs A>G 1960s | -0.387 | 0.538 | -0.719 | 0.472 | 0.729 |
| Neoascochyta | A>G 1900s vs G>F 1900s | -1.566 | 0.371 | -4.219 | <0.001*** | 0.001 |
| Neoascochyta | A>G 1900s vs G>F 1960s | -1.479 | 0.388 | -3.807 | <0.001*** | 0.005 |
| Neoascochyta | A>G 1960s vs G>F 1900s | -1.316 | 0.404 | -3.26 | 0.001*** | 0.005 |
| Neoascochyta | A>G 1960s vs G>F 1960s | -1.081 | 0.42 | -2.576 | 0.01** | 0.065 |
| Neoascochyta | G>F 1960s vs G>F 1900s | -0.019 | 0.157 | -0.12 | 0.904 | 0.953 |
| Urocystis | SNG vs A>G 1960s | 1.196 | 0.427 | 2.804 | 0.005** | 0.066 |
| Urocystis | SNG vs A>G 1900s | 0.975 | 0.443 | 2.198 | 0.028* | 0.28 |
| Urocystis | SNG vs A>G 1800s | 0.076 | 0.26 | 0.294 | 0.769 | 0.948 |
| Urocystis | SNG vs G>F 1900s | -0.484 | 0.146 | -3.303 | 0.001*** | 0.004 |
| Urocystis | SNG vs G>F 1960s | -0.474 | 0.176 | -2.699 | 0.007** | 0.04 |
| Urocystis | A>G 1800s vs A>G 1900s | 0.911 | 0.479 | 1.902 | 0.057. | 0.312 |
| Urocystis | A>G 1800s vs A>G 1960s | 1.254 | 0.463 | 2.706 | 0.007** | 0.089 |
| Urocystis | A>G 1800s vs G>F 1900s | -0.47 | 0.233 | -2.019 | 0.043* | 0.169 |
| Urocystis | A>G 1800s vs G>F 1960s | -0.33 | 0.252 | -1.308 | 0.191 | 0.531 |
| Urocystis | A>G 1900s vs A>G 1960s | 0.268 | 0.587 | 0.457 | 0.648 | 0.895 |
| Urocystis | A>G 1900s vs G>F 1900s | -1.403 | 0.428 | -3.275 | 0.001*** | 0.014 |
| Urocystis | A>G 1900s vs G>F 1960s | -1.329 | 0.439 | -3.025 | 0.002** | 0.045 |
| Urocystis | A>G 1960s vs G>F 1900s | -1.807 | 0.411 | -4.399 | <0.001*** | 0 |
| Urocystis | A>G 1960s vs G>F 1960s | -1.585 | 0.422 | -3.755 | <0.001*** | 0.003 |
| Urocystis | G>F 1960s vs G>F 1900s | -0.006 | 0.133 | -0.046 | 0.963 | 0.963 |
| Venturia | SNG vs A>G 1960s | -0.19 | 0.189 | -1.005 | 0.315 | 0.683 |
| Venturia | SNG vs A>G 1900s | 0.339 | 0.256 | 1.326 | 0.185 | 0.656 |
| Venturia | SNG vs A>G 1800s | -0.153 | 0.193 | -0.793 | 0.428 | 0.802 |
| Venturia | SNG vs G>F 1900s | 1.537 | 0.255 | 6.02 | <0.001*** | 0 |
| Venturia | SNG vs G>F 1960s | 0.985 | 0.241 | 4.085 | <0.001*** | 0.002 |
| Venturia | A>G 1800s vs A>G 1900s | 0.505 | 0.271 | 1.864 | 0.062. | 0.312 |
| Venturia | A>G 1800s vs A>G 1960s | 0.096 | 0.209 | 0.461 | 0.645 | 0.855 |
| Venturia | A>G 1800s vs G>F 1900s | 1.779 | 0.27 | 6.587 | <0.001*** | 0 |
| Venturia | A>G 1800s vs G>F 1960s | 1.358 | 0.257 | 5.288 | <0.001*** | 0 |
| Venturia | A>G 1900s vs A>G 1960s | -0.483 | 0.269 | -1.8 | 0.072. | 0.367 |
| Venturia | A>G 1900s vs G>F 1900s | 1.253 | 0.318 | 3.933 | <0.001*** | 0.002 |
| Venturia | A>G 1900s vs G>F 1960s | 0.766 | 0.307 | 2.491 | 0.013* | 0.124 |
| Venturia | A>G 1960s vs G>F 1900s | 1.599 | 0.268 | 5.972 | <0.001*** | 0 |
| Venturia | A>G 1960s vs G>F 1960s | 1.26 | 0.254 | 4.955 | <0.001*** | 0 |
| Venturia | G>F 1960s vs G>F 1900s | 0.555 | 0.307 | 1.81 | 0.07. | 0.374 |

**Table S14**: Spearman correlation coefficients between plant and soil bacterial diversity and soil-borne fungal pathogen richness and community composition. Correlations are based on current grassland sites (i.e. semi-natural grasslands and current grasslands with a history of arable land use).

| ***Fungal pathogen*** | ***Property*** | ***r*** | ***p-value*** |
| --- | --- | --- | --- |
| Richness | Plant richness | -0.17 | 0.165 |
| Richness | Plant community composition (PC1) | -0.28 | 0.019* |
| Richness | Plant community composition (PC2) | -0.21 | 0.079 |
| Richness | Bacterial community composition (PC1) | -0.17 | 0.162 |
| Richness | Bacterial community composition (PC2) | -0.27 | 0.026* |
| Community composition (PC1) | Plant richness | -0.10 | 0.416 |
| Community composition (PC1) | Plant community composition (PC1) | -0.31 | 0.009** |
| Community composition (PC1) | Plant community composition (PC2) | -0.07 | 0.585 |
| Community composition (PC1) | Bacterial community composition (PC1) | -0.05 | 0.709 |
| Community composition (PC1) | Bacterial community composition (PC2) | -0.38 | 0.001** |
| Community composition (PC2) | Plant richness | 0.11 | 0.416 |
| Community composition (PC2) | Plant community composition (PC1) | 0.17 | 0.171 |
| Community composition (PC2) | Plant community composition (PC2) | -0.02 | 0.85 |
| Community composition (PC2) | Bacterial community composition (PC1) | 0.16 | 0.189 |
| Community composition (PC2) | Bacterial community composition (PC2) | 0.09 | 0.482 |

| ***Response variable*** | ***Predictor variable*** | ***r*** | ***p-value*** |
| --- | --- | --- | --- |
| Richness | Plant richness | -0.17 | 0.165 |
| Richness | Plant community composition (PC1) | -0.28 | 0.019* |
| Richness | Plant community composition (PC2) | -0.21 | 0.079 |
| Richness | Bacterial community composition (PC1) | -0.17 | 0.162 |
| Richness | Bacterial community composition (PC2) | -0.27 | 0.026* |
| Community composition (PC1) | Plant richness | -0.10 | 0.416 |
| Community composition (PC1) | Plant community composition (PC1) | -0.31 | 0.009** |
| Community composition (PC1) | Plant community composition (PC2) | -0.07 | 0.585 |
| Community composition (PC1) | Bacterial community composition (PC1) | -0.05 | 0.709 |
| Community composition (PC1) | Bacterial community composition (PC2) | -0.38 | 0.001** |
| Community composition (PC2) | Plant richness | 0.11 | 0.416 |
| Community composition (PC2) | Plant community composition (PC1) | 0.17 | 0.171 |
| Community composition (PC2) | Plant community composition (PC2) | -0.02 | 0.85 |
| Community composition (PC2) | Bacterial community composition (PC1) | 0.16 | 0.189 |
| Community composition (PC2) | Bacterial community composition (PC2) | 0.09 | 0.482 |

**Table S15:** Network properties of microbial (fungi, bacteria) co-occurrence networks.

|  | ***A>G 1960s*** | ***A>G 1900s*** | ***SNG*** | ***G>F 1960s*** | ***G>F 1900s*** |
| --- | --- | --- | --- | --- | --- |
| ***Fungi*** |  |  |  |  |  |
| Clustering coefficient | 0.446 | 0.580 | 0.328 | 0.361 | 0.267 |
| Modularity | 0.536 | 0.735 | 0.660 | 0.631 | 0.733 |
| Network size | 3215 | 1179 | 755 | 802 | 405 |
| Average path length | 4.01 | 6.05 | 5.15 | 4.99 | 5.65 |
| Positive:Negative correlations | 1 | 1 | 0.995 | 0.993 | 0.985 |
| ***Bacteria*** |  |  |  |  |  |
| Clustering coefficient | 0.582 | 0.548 | 0.555 | 0.593 | 0.524 |
| Modularity | 0.268 | 0.558 | 0.257 | 0.209 | 0.354 |
| Network size | 29300 | 7205 | 13660 | 28754 | 16627 |
| Average path length | 2.39 | 3.51 | 2.74 | 2.32 | 2.431 |
| Positive:Negative correlations | 0.821 | 0.997 | 0.636 | 0.685 | 0.748 |

***References***

Abarenkov, K., Nilsson, R.H., Larsson, K.-H., Taylor, A.F.S., May, T.W., Frøslev, T.G., *et al.* (2024). The UNITE database for molecular identification and taxonomic communication of fungi and other eukaryotes: sequences, taxa and classifications reconsidered. *Nucleic Acids Research*, 52, D791–D797.

Blanchet, F.G., Cazelles, K. & Gravel, D. (2020). Co-occurrence is not evidence of ecological interactions. *Ecology Letters*, 23, 1050–1063.

Cousins, S.A.O. (2001). Analysis of land-cover transitions based on 17th and 18th century cadastral maps and aerial photographs. *Landscape Ecology*, 16, 41–54.

Csárdi, G., Nepusz, T., Traag, V., Horvát, S., Zanini, F., Noom, D., *et al.* (2025). igraph: Network Analysis and Visualization in R.

Edgar, R.C. (2013). UPARSE: highly accurate OTU sequences from microbial amplicon reads. *Nat Methods*, 10, 996–998.

Edgar, R.C., Haas, B.J., Clemente, J.C., Quince, C. & Knight, R. (2011). UCHIME improves sensitivity and speed of chimera detection. *Bioinformatics*, 27, 2194–2200.

Eriksson, O. & Cousins, S.A.O. (2014). Historical Landscape Perspectives on Grasslands in Sweden and the Baltic Region. *Land*, 3, 300–321.

Gustavsson, E., Lennartsson, T. & Emanuelsson, M. (2007). Land use more than 200   years ago explains current grassland plant diversity in a Swedish agricultural landscape. *Biological Conservation*, 138, 47–59.

Ihrmark, K., Bödeker, I.T.M., Cruz-Martinez, K., Friberg, H., Kubartova, A., Schenck, J., *et al.* (2012). New primers to amplify the fungal ITS2 region – evaluation by 454-sequencing of artificial and natural communities. *FEMS Microbiology Ecology*, 82, 666–677.

Kõljalg, U., Nilsson, H.R., Schigel, D., Tedersoo, L., Larsson, K.-H., May, T.W., *et al.* (2020). The Taxon Hypothesis Paradigm—On the Unambiguous Detection and Communication of Taxa. *Microorganisms*, 8, 1910.

Kurtz, Z.D., Müller, C.L., Miraldi, E.R., Littman, D.R., Blaser, M.J. & Bonneau, R.A. (2015). Sparse and Compositionally Robust Inference of Microbial Ecological Networks. *PLOS Computational Biology*, 11, e1004226.

Özkurt, E., Fritscher, J., Soranzo, N., Ng, D.Y.K., Davey, R.P., Bahram, M., *et al.* (2022). LotuS2: an ultrafast and highly accurate tool for amplicon sequencing analysis. *Microbiome*, 10, 176.

Quast, C., Pruesse, E., Yilmaz, P., Gerken, J., Schweer, T., Yarza, P., *et al.* (2013). The SILVA ribosomal RNA gene database project: improved data processing and web-based tools. *Nucleic Acids Research*, 41, D590–D596.

Tedersoo, L. & Lindahl, B. (2016). Fungal identification biases in microbiome projects. *Environmental Microbiology Reports*, 8, 774–779.

Walters, W., Hyde, E.R., Berg-Lyons, D., Ackermann, G., Humphrey, G., Parada, A., *et al.* (2015). Improved Bacterial 16S rRNA Gene (V4 and V4-5) and Fungal Internal Transcribed Spacer Marker Gene Primers for Microbial Community Surveys. *mSystems*, 1.

Weiss, S., Van Treuren, W., Lozupone, C., Faust, K., Friedman, J., Deng, Y., *et al.* (2016). Correlation detection strategies in microbial data sets vary widely in sensitivity and precision. *The ISME Journal*, 10, 1669–1681.
